# MiR144/451 Expression Is Repressed by RUNX1 During Megakaryopoiesis and Disturbed by RUNX1/ETO

**DOI:** 10.1371/journal.pgen.1005946

**Published:** 2016-03-18

**Authors:** Nicole Kohrs, Stephan Kolodziej, Olga N. Kuvardina, Julia Herglotz, Jasmin Yillah, Stefanie Herkt, Alexander Piechatzek, Gabriela Salinas Riester, Thomas Lingner, Christian Wichmann, Halvard Bonig, Erhard Seifried, Uwe Platzbecker, Hind Medyouf, Manuel Grez, Jörn Lausen

**Affiliations:** 1 Georg-Speyer-Haus, Institute for Tumorbiology and Experimental Therapy, Frankfurt, Germany; 2 Heinrich-Pette-Institute, Leibniz Institute for Experimental Virology, Hamburg, Germany; 3 Medical-University Goettingen, Transcriptome Analysis Laboratory, Goettingen, Germany; 4 Department of Transfusion Medicine, Cell Therapeutics and Hemostaseology, Ludwig-Maximilian University Hospital, Munich, Germany; 5 Institute for Transfusion Medicine and Immunohematology, Johann-Wolfgang-Goethe University and German Red Cross Blood Service, Frankfurt am Main, Germany; 6 Department of Hematology, Medical Clinic and Polyclinic I, University Hospital Carl Gustav Carus, Dresden, Germany; University of Maryland Medical School, UNITED STATES

## Abstract

A network of lineage-specific transcription factors and microRNAs tightly regulates differentiation of hematopoietic stem cells along the distinct lineages. Deregulation of this regulatory network contributes to impaired lineage fidelity and leukemogenesis. We found that the hematopoietic master regulator RUNX1 controls the expression of certain microRNAs, of importance during erythroid/megakaryocytic differentiation. In particular, we show that the erythorid miR144/451 cluster is epigenetically repressed by RUNX1 during megakaryopoiesis. Furthermore, the leukemogenic RUNX1/ETO fusion protein transcriptionally represses the miR144/451 pre-microRNA. Thus RUNX1/ETO contributes to increased expression of miR451 target genes and interferes with normal gene expression during differentiation. Furthermore, we observed that inhibition of RUNX1/ETO in Kasumi1 cells and in RUNX1/ETO positive primary acute myeloid leukemia patient samples leads to up-regulation of miR144/451. RUNX1 thus emerges as a key regulator of a microRNA network, driving differentiation at the megakaryocytic/erythroid branching point. The network is disturbed by the leukemogenic RUNX1/ETO fusion product.

## Author Summary

The regulatory network between transcription factors, epigenetic cofactors and microRNAs is decisive for normal hematopoiesis. The transcription factor RUNX1 is important for the establishment of a megakaryocytic gene expression program and the concomitant repression of erythroid genes during megakaryocytic differentiation. Gene regulation by RUNX1 is frequently disturbed by mutations and chromosomal translocations, such as the t(8;21) translocation, which gives rise to the leukemogenic RUNX1/ETO fusion protein. We found that RUNX1 regulates microRNAs, which are of importance at the megakaryocytic/erythroid branching point. Specifically, RUNX1 down-regulates expression of the microRNA cluster miR144/451 during megakaryocytic differentiation by changing the epigenetic histone modification pattern at the locus. We could show that miR451, one of the micorRNAs of the miR144/451 cluster, supports erythroid differentiation. We found that expression of miR451 is repressed by the RUNX1/ETO fusion protein, resulting in up regulation of miR451 target genes. Our data support the notion that RUNX1 suppresses the erythroid gene expression program including the erythroid microRNA miR451 and that the RUNX1/ETO fusion protein interferes with normal gene regulation by RUNX1.

## Introduction

The transcription factor RUNX1 (or AML1, acute myeloid leukemia 1) is a critical regulator of embryonic and adult hematopoiesis (reviewed in [1–3]). Alteration in RUNX1 due to chromosomal translocations and mutations are causally connected to the onset of acute myeloid leukemia in humans [4]. RUNX1 possesses a pivotal role in myeloid lineage differentiation, is a crucial regulator of gene expression at the megakaryocytic/erythroid branching [5–7] and is down-regulated during erythropoiesis [8,9]. We recently reported that RUNX1 inhibits erythroid differentiation by repressing the erythroid gene expression program [5]. During megakaryopoiesis sustained RUNX1 expression represses the erythroid master regulator KLF1 [5].

RUNX1 is involved in the t(8;21) chromosomal translocation found in approximately 15% of acute myeloid leukemia cases, where the DNA binding runt homology domain (RHD) of RUNX1 and almost the entire ETO (MTG8) protein are fused [10–12]. The resulting RUNX1/ETO fusion protein can act as a constitutive transcriptional repressor, which occupies RUNX1 binding sites [13,14]. RUNX1/ETO does not induce leukemia on its own [15–17]. However, it may contribute to outgrowth of a pre-leukemic clone, which by gathering additional mutations evolves into leukemia [18]. A shorter variant of RUNX1/ETO, RUNX/ETO9a, lacking the C-terminal domain of ETO induces leukemia in murine bone-marrow transplantation models [19–21]. Similar to RUNX1, full length RUNX1/ETO has an inhibitory effect on erythropoiesis [5,22]. Furthermore, both RUNX1 and its leukemic fusion protein RUNX1/ETO influence expression of a number of microRNAs in normal differentiation and leukemia [23]. Thus, we posit that the disturbance of lineage differentiation such as erythropoiesis by RUNX1/ETO might be mediated through alterations of microRNA expression, in addition to the disturbance of transcriptional networks [24]. Because RUNX1 inhibits erythroid gene expression [5] and RUNX1/ETO interferes with erythroid differentiation [22], we were interested in downstream microRNAs at the megakaryocytic/erythroid bifurcation.

The microRNAs miR144 and miR451 are up regulated during erythroid differentiation [25–31]. MiR144 and miR451 are transcribed as one pri-microRNA (referred to as miR144/451), which is regulated by the activity of the transcription factor GATA1 [26]. Interestingly, maturation of miR144 and miR451 are distinct, as miR451 is processed dicer independently [32–34]. Knock-down experiments have established a positive effect of mature miR451 on erythropoiesis [26,27,29,35,36] but little effect of mature miR144 [26,37].

In this study, we identified microRNAs downstream of RUNX1 in human hematopoietic cells. We found that the erythroid specific miR144/451 cluster is transcriptionally regulated by RUNX1 and TAL1, in addition to GATA1 [26]. We show that RUNX1 binds to the promoter of miR144/451 and is an epigenetic repressor of miR144/451 expression during megakaryocytic differentiation. Thus RUNX1 contributes to the down-regulation of the erythroid gene expression program by repressing miR451 transcription. Furthermore, the leukemogenic RUNX1/ETO fusion protein interferes with miR144/451 expression and disturbs miR451 function. This RUNX1/ETO mediated repression of miR451 activity can be reversed by inhibition of RUNX1/ETO.

## Results

### RUNX1 controls a network of hematopoietic microRNAs

**Fig 1:**
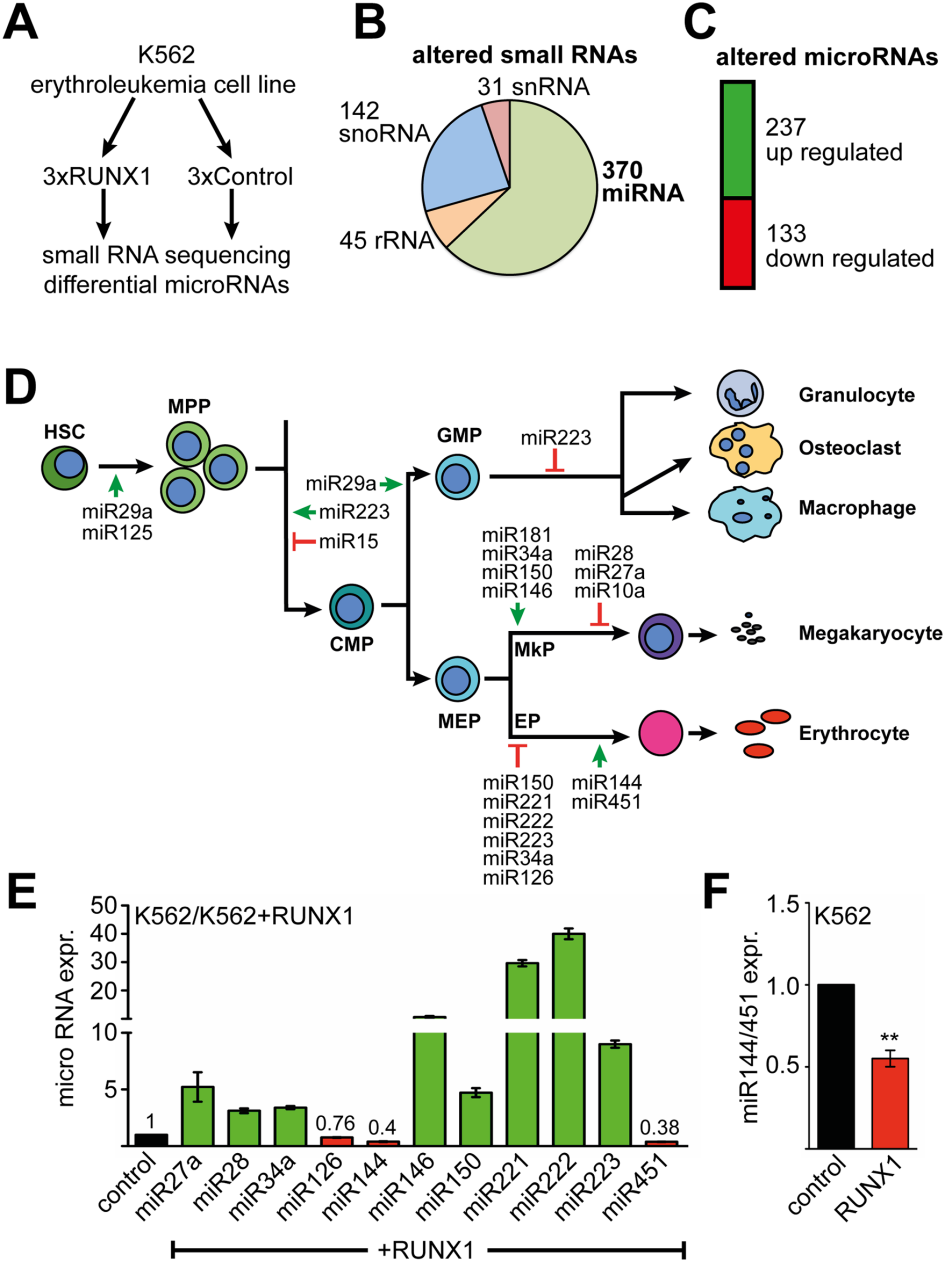
Identification of RUNX1 regulated microRNAs. **(A)** Schematic representation of the experimental setup. K562 cells were transduced with RUNX1 expression vector or empty vector control. The transductions were performed in independent triplicates and differentially expressed microRNAs were determined by small-RNA sequencing. **(B)** 588 small RNAs were differentially expressed upon RUNX1 expression in K562 cells. 31 were snRNAs (small nuclear RNA), 45 rRNAs (ribosomal RNA), 142 snoRNAs (small nucleolar RNA) and 370 microRNAs. RNAs were included if they displayed an at least -0.5 or +0.5 log2-fold change and a P-value <0.05. **(C)** Of the 370 identified microRNAs, 237 were up-regulated and 133 down-regulated upon RUNX1 expression. **(D)** Schematic representation of hematopoiesis from the stem cell throughout the myeloid lineage. Those microRNAs are shown, which were altered upon RUNX1 over-expression. The green arrow indicates a positive role and the red blunted arrow indicates a negative role in differentiation according to published work. HSC: hematopoietic stem cell, MPP: multipotent progenitor, CMP: common myeloid progenitor, GMP: granulocyte monocyte progenitor, MEP: megakaryocyte erythrocyte progenitor, MkP: megakaryocyte progenitor, EP: erythrocyte progenitor. **(E)** Independent evaluation of microRNA expression. A subset of mature microRNAs influenced by RUNX1 and with a role in myeloid differentiation identified by RNA-sequencing, was tested by q-RT-PCR. Q-RT-PCR values are given as relative expression of RUNX1 transduced K562 cells, compared to K562 cells transduced with empty vector. Values were normalised to RNU6-2 expression. The error bars give the standard deviation from four independent determinations. All values were significantly different from the control according to Student’s t test P <0.05. **(F)** RUNX1 over-expression leads to a decrease of miR144/451 (pri-microRNA) transcript. Q-RT-PCR values are shown as fold expression compared to empty vector transduced K562 cells. Error bars represent the standard deviation from four independent determinations. The P-value was calculated using Student’s t test. **P <0.01.

We previously showed that RUNX1 represses the erythroid gene expression program during megakaryocytic differentiation [5]. Thus we wanted to examine specifically which microRNAs contribute to the biological function of RUNX1 at this differentiation point. To determine, which microRNAs are regulated by RUNX1 at the megakaryocytic/erythroid branching we used K562 erythroleukemia cells, as they have the potential to differentiate towards the erythroid or megakaryocytic lineage. We performed small-RNA sequencing upon over-expression of RUNX1 in K562 cells (Fig 1A) and observed 588 altered small RNAs (Fig 1B and S1 File). Of these altered small RNAs, 31 were snRNAs (small nuclear RNA), 45 rRNAs (ribosomal RNA), 142 snoRNAs (small nucleolar RNA) and 370 microRNAs (Fig 1B). A total of 237 microRNAs were up-regulated and 133 microRNAs were down-regulated upon RUNX1 over-expression (Fig 1C). Because RUNX1 is an important transcription factor for megakaryocytic and erythroid differentiation we were especially interested in microRNAs connected to RUNX1 with a known role during erythroid or megakaryocytic differentiation (Fig 1D) [38,39]. Subsequently, we analysed the relative expression of a subset of these microRNAs by q-RT-PCR detecting the mature microRNAs in parental versus RUNX1 expressing cells (Fig 1E). All microRNAs measured by q-RT-PCR were regulated in the same direction as measured by RNAseq, except for miR144 (S1 Fig). In line with published data miR27a, miR126, miR222, miR223 were sensitive to changes in RUNX1 expression [40–44]. The expression of most differentially expressed microRNAs was up-regulated, except miR126, miR144 and miR451. MiR144 and miR451 are transcribed as one pri-microRNA, driven by a shared promoter. Because RUNX1 is a transcriptional regulator we analysed expression of the transcriptionally regulated pri-micro RNA and found that expression of the pri-microRNA (referred to as miR144/451) was decreased in RUNX1 over-expressing K562 cells (Fig 1F and S2 Fig).

### RUNX1 and TAL1 regulate miR144/451

**Fig 2:**
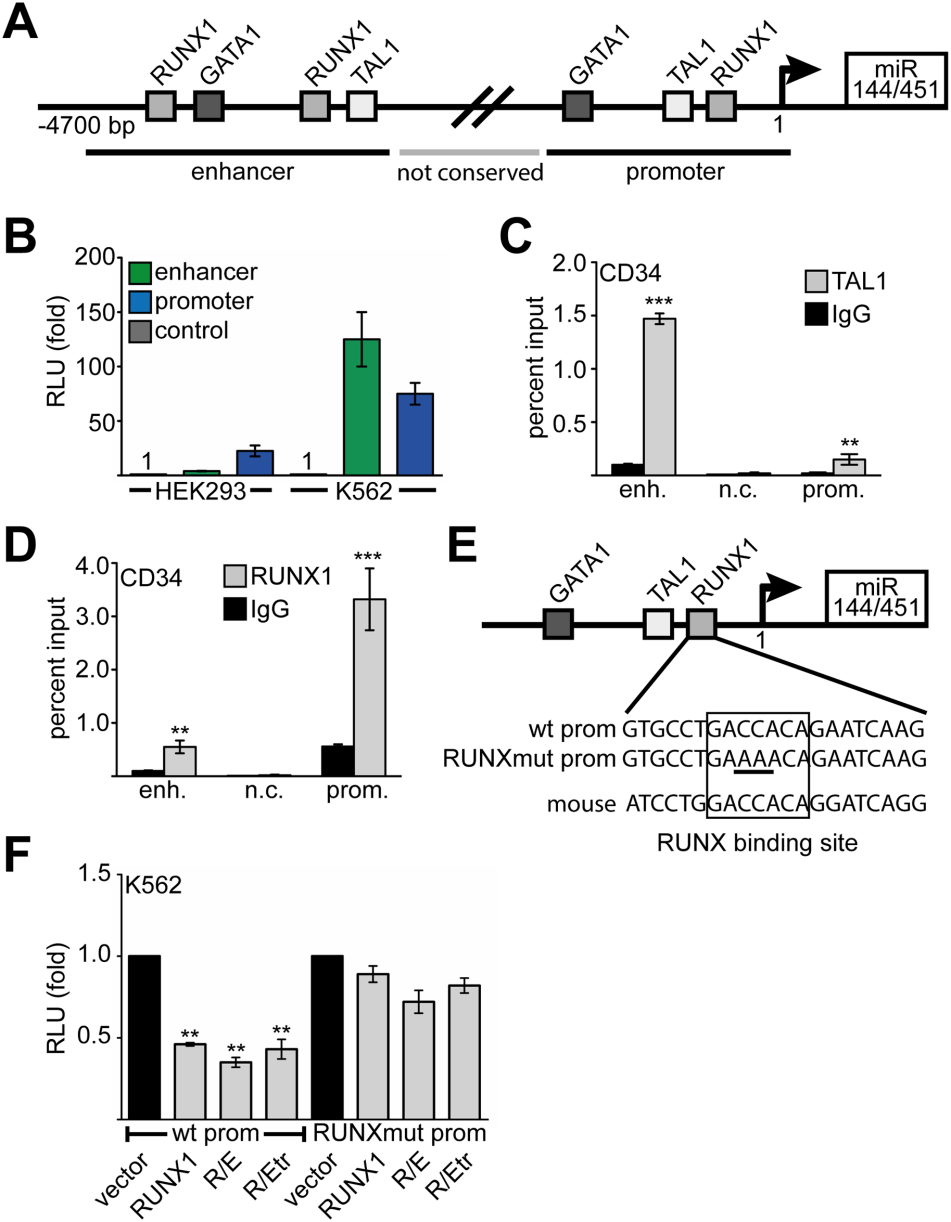
Regulation of miR144/451 expression. **(A)** Schematic representation of the miR144/451 locus. The first 4700 bp of the 5’-region of miR144/451 are shown. Binding sites for hematopoietic transcription factors are found about 4500 bp upstream (enhancer) and in the proximal promoter region (promoter). A less conserved region (not conserved, n.c.) separates these regions. **(B)** Luciferase reporter gene experiment with constructs harbouring the enhancer or the promoter region of the miR144/451 cluster. Reporter constructs were transfected into HEK293 cells or K562 cells, respectively. Relative light units (RLU) are given as fold induction compared to values gathered with empty luciferase vector. Luciferase values were normalised for transfection variations by measuring the activity of a cotransfected beta-galactosidase expression vector. Error bars indicate the standard deviation from six independent transfections and measurements. **(C)** ChIP assay in primary hCD34+ cells shows binding of TAL1 predominantly to the enhancer region (enh.) as opposed to the promoter region (prom.). **(D)** ChIP assay in hCD34+ cells shows binding of RUNX1 to the promoter region (prom.) but only little to the enhancer region (enh.). **(E)** The human miR144/451 promoter contains a conserved binding site for RUNX1. The RUNX1 binding site was mutated by changing two base pairs in the core RUNX1 site. **(F)** Luciferase reporter assay using the wild type (wt prom) and the mutated (RUNXmut) miR144/451 promoters, respectively. Transfection of RUNX1, the RUNX1/ETO (R/E) fusion protein or the truncated RUNX1/ETO (R/Etr) repressed wild type (wt prom), but not the mutated promoter (RUNX1mut prom). Values are presented as fold change compared to the relative light units gathered upon transfection of the reporter gene and empty expression vector. Luciferase values were normalised for transfection variations by measuring the activity of a cotransfected beta-galactosidase expression vector. Error bars represent the standard deviation from six independent transfections and measurements. The P-value was calculated using Student’s t test. **P <0.01.

To evaluate if RUNX1 directly influences miR144/451 expression, we analysed the 5’-region of the miR144/451 locus *in-silico*, using the human genome browser [45]. Two regions in the 5’-area of miR144/451 display a high degree of homology between species (Fig 2A and S3 Fig), these areas are separated by a region of lower sequence homology (Fig 2A, not conserved or n.c.). We identified potential RUNX1, TAL1 and GATA1 binding sites close to the transcriptional start site and at an upstream (enhancer) region using TESS [46]. GATA1 binding at the enhancer region had previously been demonstrated [26]. The enhancer and promoter regions displayed high activity in a reporter gene assay in hematopoietic K562 cells compared to embryonic kidney HEK293 cells (Fig 2B), indicating that the miR144/451 regulatory elements are active in hematopoietic cells. By performing systematic over-expression and knock-down experiments of RUNX1, TAL1 and GATA1 in K562 cells, we found that RUNX1 repressed miR144/451 expression, while TAL1 and GATA1 activated it (S2 Fig).

Chromatin immunoprecipitation (ChIP) in primary hCD34+ and K562 cells revealed that TAL1 mainly binds to the enhancer region of miR144/451 and to a less degree at the promoter region (Fig 2C and S4 Fig). In contrast, RUNX1 binding was mainly detected at the promoter region of miR144/451 (Fig 2D). This promoter region harbours a RUNX1 binding site, which is also present in the mouse promoter sequence (Fig 2E). We tested the influence of RUNX1 on the miR144/451 promoter in a luciferase reporter assay and found that RUNX1 repressed miR144/451 promoter activity (Fig 2F). Interestingly, the oncogenic RUNX1 t(8;21) fusion proteins RUNX1/ETO (R/E) and also the truncated RUNX1/ETO (R/Etr) repressed miR144/451 promoter activity (Fig 2F, wt prom). In contrast, neither RUNX1, nor R/E or R/Etr were able to repress the promoter when the RUNX1 binding site was mutated (Fig 2F, RUNXmut prom, compare Fig 2E). This indicates that both RUNX1 and its RUNX1/ETO leukemogenic fusion protein repress the miR144/451 promoter by directly binding to the RUNX1 binding site.

### RUNX1 binding is associated with repression of miR144/451 expression

**Fig 3:**
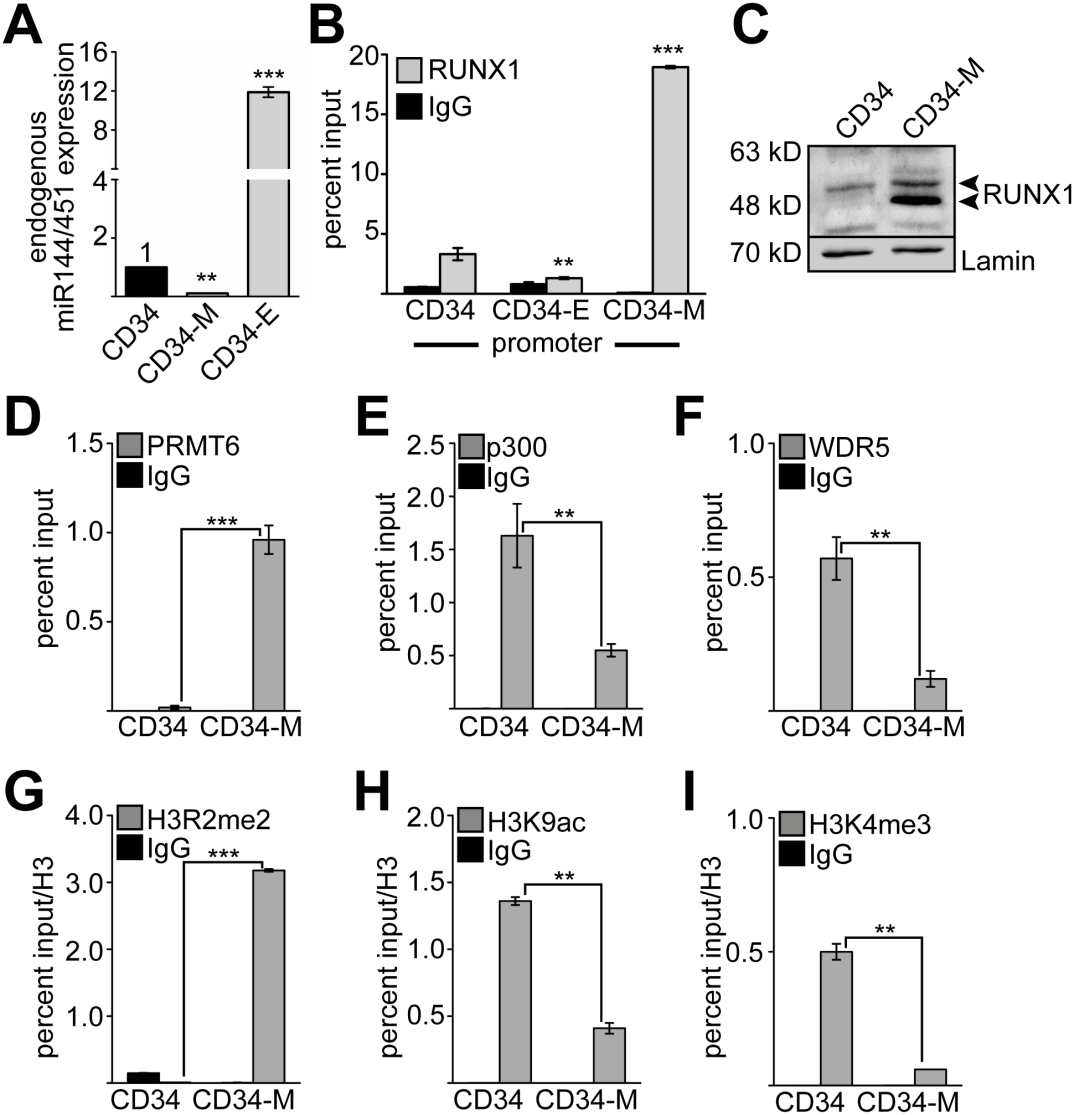
MiR144/451 expression is epigenetically repressed during megakaryocytic differentiation. **(A)** MiR144/451 expression is decreased upon megakaryocytic differentiation and increased upon erythroid expression of primary hCD34+ cells. Differentiation was done for 6 days (see S7 Fig). Expression of the endogenous pri-microRNA was measured by q-RT-PCR. **(B)** ChIP assay analysis of RUNX1 binding to the miR144/451 promoter during erythroid (CD34-E) or megakaryocytic (CD34-M) differentiation of hCD34+ (CD34) cells. **(C)** Analysis of RUNX1 protein abundance in hCD34+ cells and upon megakaryocytic differentiation (CD34-M) by Western blot using a RUNX1 antibody and a lamin antibody as loading control. **(D-I)** Cofactor and histone modification changes at the promoter of miR144/451 during megakaryocytic differentiation (CD34-M) using ChIP analysis in hCD34+ cells. **(D-F)** ChIP analysis reveals altered binding of PRMT6, p300 and WDR5 to the miR144/451 promoter upon megakaryocytic differentiation. **(G-I)** ChIP reveals altered H3R2me2, H3K9ac and H3K4me3 at the miR144/451 promoter upon megakaryocytic differentiation. **(D-I)** Q-PCR values are given as percent input. Histone modification ChIP values were corrected by a Histone 3 ChIP for nucleosome density. The error bars represent the standard deviation from at least four independent determinations. The P-values were calculated using Student’s t test. **P <0.01, ***P <0.001.

MiR144/451 expression is down-regulated during megakaryocytic and up-regulated during erythroid differentiation of hCD34+ (Fig 3A) and K562 cells (S5 Fig). We found that RUNX1 binding at the promoter region of miR144/451 was reduced upon erythroid differentiation and strongly up-regulated upon megakaryocytic differentiation of hCD34+ cells (Fig 3B) and K562 cells (S5 Fig). Concomitant to the enhanced RUNX1 binding to the miR144/451 promoter, RUNX1 expression increased upon megakaryocytic differentiation at the protein level in CD34 cells (Fig 3C) and K562 cells (S5 Fig). Interestingly, upon megakaryocytic differentiation of primary hCD34+ cells an additional RUNX1 band appeared. These RUNX1 isoforms have been described before as the RUNX1b (upper band) and RUNX1a (lower band) isoforms [47]. Increased RUNX1 binding at the promoter region during megakaryocytic differentiation was accompanied by an increase of the RUNX1 associated corepressor protein PRMT6 [6,7] (Fig 3D). At the same time binding of the coactivators p300 and WDR5 decreased upon megakaryocytic differentiation in hCD34+ and K562 cells (Fig 3E and 3F). Concomitantly, the repressive H3R2me2 histone modification mark increased, while the activating modification marks, H3K9ac and H3K4me3, were significantly decreased in hCD34+ and K562 cells (Fig 3G, 3H and 3I and S4 Fig). These data are in agreement with a repressive role of RUNX1 on erythroid genes during megakaryocytic differentiation [5].

### MiR451 influences erythroid differentiation of primary hCD34+ cells

**Fig 4:**
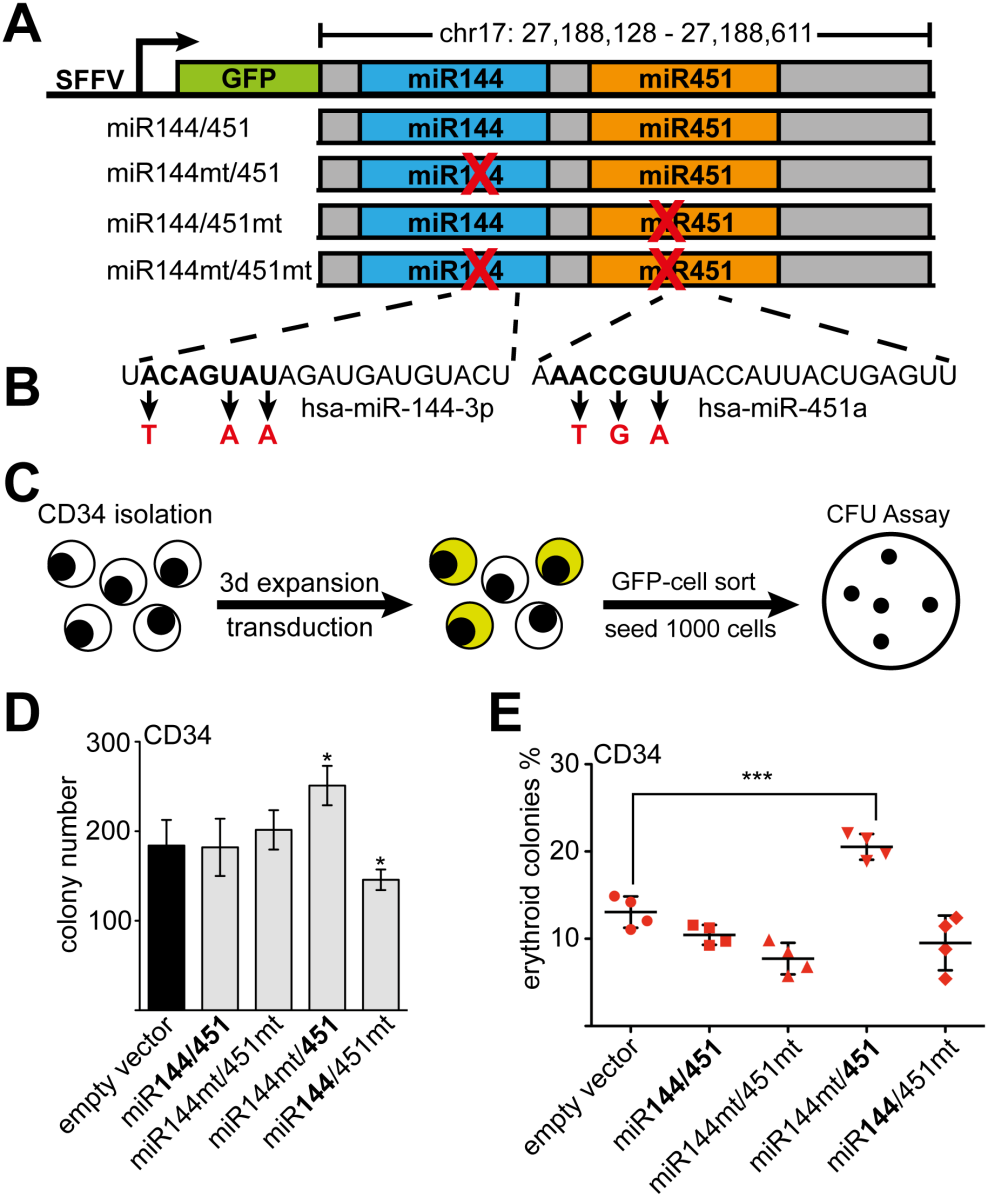
MiR451 increases erythroid differentiation. **(A)** Schematic representation of miR144/451 expression constructs. The genomic sequence of human miR144/451 was cloned into the LEGO vector [48] behind the GFP expression cassette, which is driven by a spleen focus-forming virus promoter. The wild type and mutant constructs are shown, with the X marking the mutational site. **(B)** The mutations introduced in the seed sequences of miR144 and miR451 are indicated. **(C)** A colony formation assay was performed with sorted GFP-positive transduced hCD34+ cells. **(D)** The total number of colonies in the colony formation assay is shown. Colonies were counted on day 12. **E)** The frequency (%) of erythroid colonies among total colonies in the colony formation assay is shown. Error bars represent the standard deviation of four independent determinations. *P-value <0.05, ***P-value <0.001 according to Student’s t test.

A positive role of miR451 in erythropoiesis in cell lines, zebrafish and mouse models was demonstrated [26,27,29,35]. This notion is also strengthened by our observation that the mature miR451 is more abundant than mature miR144 upon erythroid differentiation (S6 Fig). To test the influence of the single microRNAs in our differentiation system with primary hCD34+ cells (S7 Fig), we constructed miR144/451 expression vectors (Fig 4A). These contained the genomic region of the miR144/451 locus including the entire pri-microRNA (S8 Fig). The different constructs expressed either the wild type miR144/451 or versions, in which the seed sequence of miR144 or miR451 was mutated, respectively (Fig 4B). We transduced human CD34+ stem-/progenitor cells with the given constructs and performed colony-forming assays (Fig 4C and S7 Fig). The total number of colonies was increased upon over-expression of miR451 and reduced with miR144 compared to the empty vector control (Fig 4D). Expression of the wild type miR144/451 construct did not alter erythroid colony formation, whereas expression of the double mutant slightly reduced erythroid colony number. MiR451 (miR144mt/451) increased erythroid colony number, whereas miR144 (miR144/451mt) increased CFU-G number, but had no effect on erythroid colonies (Fig 4E). In context of the wild type miR144/451 construct miR144 seemed to counteract miR451 influence on erythopoiesis in this assay (Fig 4E). Taken together, our data confirm that miR451 positively influences erythroid differentiation of primary hCD34+ progenitor cells.

### RUNX1/ETO represses miR144/451 expression

**Fig 5:**
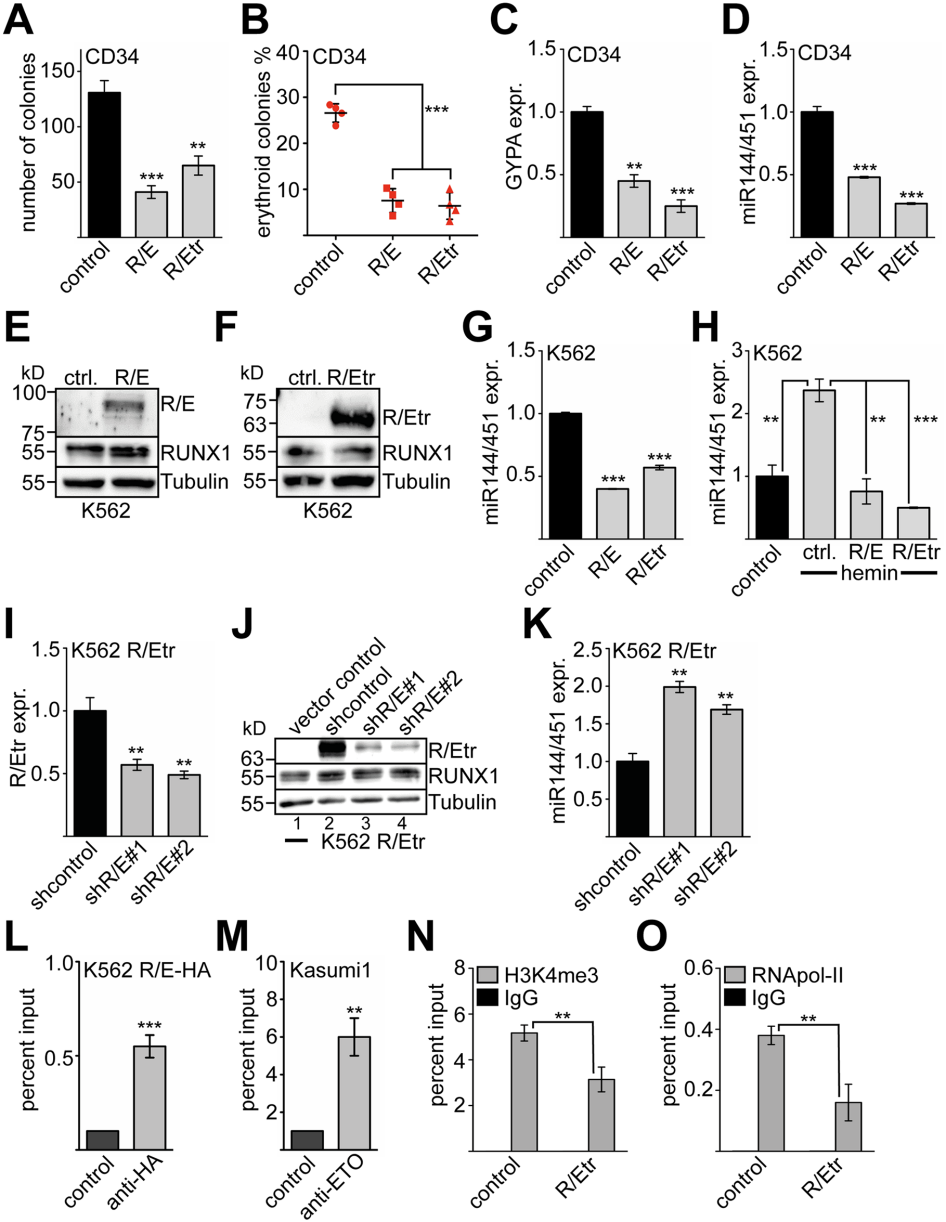
RUNX1/ETO inhibits miR144/451 expression during erythroid differentiation. **(A)** Colony formation assay with sorted GFP-positive transduced hCD34+ cells shows that full-length RUNX1/ETO (R/E) and RUNX1/ETOtr (R/Etr) inhibit colony formation. The average number of colonies per experiment is given. The error bars give the standard deviation from four independent experiments. **(B)** The percentage of erythroid colonies is reduced in a colony formation assay upon transduction of hCD34+ cells with R/E or R/Etr. Shown is the relative abundance of erythroid colonies in percent. **(C)** Expression of the erythroid marker gene GYPA is reduced on the mRNA level upon transduction of hCD34+ cell with R/E or R/Etr, as measured by q-RT-PCR. **(D)** Expression of the pri-miR144/451 is reduced upon transduction of hCD34+ cells with R/E or R/Etr, as shown by q-RT-PCR. **(E)** Expression of full length RUNX1/ETO (R/E) in transduced K562 cells. Western blot was performed with anti-HA-antibody, which detects the transduced R/E. Endogenous RUNX1 was detected with anti-RUNX1 antibody. Tubulin served as a loading control. **(F)** Expression of truncated RUNX1/ETO (R/Etr) in transduced K562 cells. Western blot was performed with anti-HA-antibody, which detects the transduced R/E. The endogenous RUNX1 was detected with an anti-RUNX1 antibody. Tubulin served as a loading control. **(G)** Expression of pri-miR144/451 is reduced upon transduction of K562 cells with R/E or R/Etr as measured by q-RT-PCR. **(H)** Induction of the miR144/451 cluster during erythroid differentiation is impaired upon transduction of K562 cells with R/E or R/Etr. Transduced K562 cells were treated with 30 μM hemin. **(I-J)** Knock-down of R/Etr expression in R/Etr-K562 cells with an shRNA targeting R/E. **(I)** The knock-down of R/Etr is shown on the mRNA level by q-RT-PCR. **(J)** The knock-down of R/Etr is shown on the protein level by Western blot using an HA-tag antibody, which detects the transduced R/Etr. An antibody against RUNX1 detected the endogenous RUNX1. Tubulin served as a loading control. **(K)** Knock-down of R/E by shRNA increases miR144/451 expression measured by q-RT-PCR. **(C-K)** Expression data were gathered by q-RT-PCR with gene specific primer pairs, values were normalised to GAPDH expression. Shown is the relative expression compared to cells transduced with empty vector or expression vectors harbouring a no-targeting shRNA, respectively. Error bars represent the standard deviation from at least four determinations. **(L)** ChIP assay in K562 cells transduced with HA-tagged RUNX1/ETO (R/E-HA) shows binding of R/E-HA to the miR144/451 promoter. For ChIP an anti-HA-tag antibody was used. **(M)** ChIP assay in Kasumi1 cells provides evidence that endogenous RUNX1/ETO binds to the miR144/451 promoter. For ChIP an anti-ETO antibody was used. **(N)** ChIP assay with K562 cells transduced with R/Etr using an anti-H3K4me3 antibody reveals reduced H3K4 methylation. **(O)** ChIP assay with K562 cells transduced with R/Etr using an anti-RNApol-II antibody reveals reduced RNA polymerase II occupancy. **(L-O)** Q-PCR was performed with primers in the promoter region of miR144/451. The P- values were calculated using Student’s t test. **P <0.01, ***P <0.001.

The RUNX1/ETO (R/E) fusion protein and its truncated variant (RUNX1/ETO9a similar to R/Etr) were shown to interfere with normal gene regulation by RUNX1 [49] and negatively impact erythroid differentiation [22,50–52]. Furthermore, RUNX1/ETO repressed the miR144/451 promoter in a reporter gene assay (compare Fig 2F). Thus, we wondered if some of the RUNX1/ETO effects might be mediated through the modulation of endogenous miR144/451. We found that RUNX1/ETO expression in primary hCD34+ cells reduced the colony number in a CFU assay (Fig 5A). Of the remaining colonies, the frequency of erythroid colonies was significantly reduced with the full-length RUNX1/ETO (R/E) and the truncated form (R/Etr) (Fig 5B). Furthermore, RUNX1/ETO expression reduced expression of the erythroid marker GYPA (Fig 5C). Notably, RUNX1/ETO inhibited the expression of miR144/451 in primary hCD34+ cells (Fig 5D). Over-expression of RUNX1/ETO full length and RUNX1/ETOtr in K562 cells (Fig 5E and 5F) left endogenous RUNX1 protein expression largely unaffected and led to inhibition of miR144/451 expression (Fig 5G) similar to hCD34+ cells. RUNX1/ETO also inhibited induction of miR144/451 expression under conditions that promote erythroid differentiation of K562 cells (Fig 5G). We then knocked-down R/Etr in K562 cells, which over-expressed R/Etr (Fig 5I and 5J) with an shRNA targeting the fusion site of RUNX1/ETO [53,54]. This did not influence endogenous RUNX1 protein expression (Fig 5J). In line with a repressive role of RUNX1/ETO, we observed increased miR144/451 expression upon RUNX1/ETOtr knock-down (Fig 5K), showing that the effect of RUNX1/ETO on miR144/451 expression is reversible. To determine if RUNX1/ETO binds endogenously to the miR144/451 promoter, we performed a ChIP-assay in K562 cells expressing an HA-tagged RUNX1/ETO and in Kasumi1 cells expressing endogenous RUNX1/ETO. In both cases, we detected RUNX1/ETO at the miR144/451 promoter (Fig 5L and 5M). Expression of RUNX1/ETOtr reduced the level of the activating histone mark H3K4me3 at the miR144/451 promoter (Fig 5N) and led to decreased occupancy of RNApol-II in K562 cells (Fig 5O).

### MiR144/451 expression can be reactivated by inhibition of RUNX1/ETO

**Fig 6:**
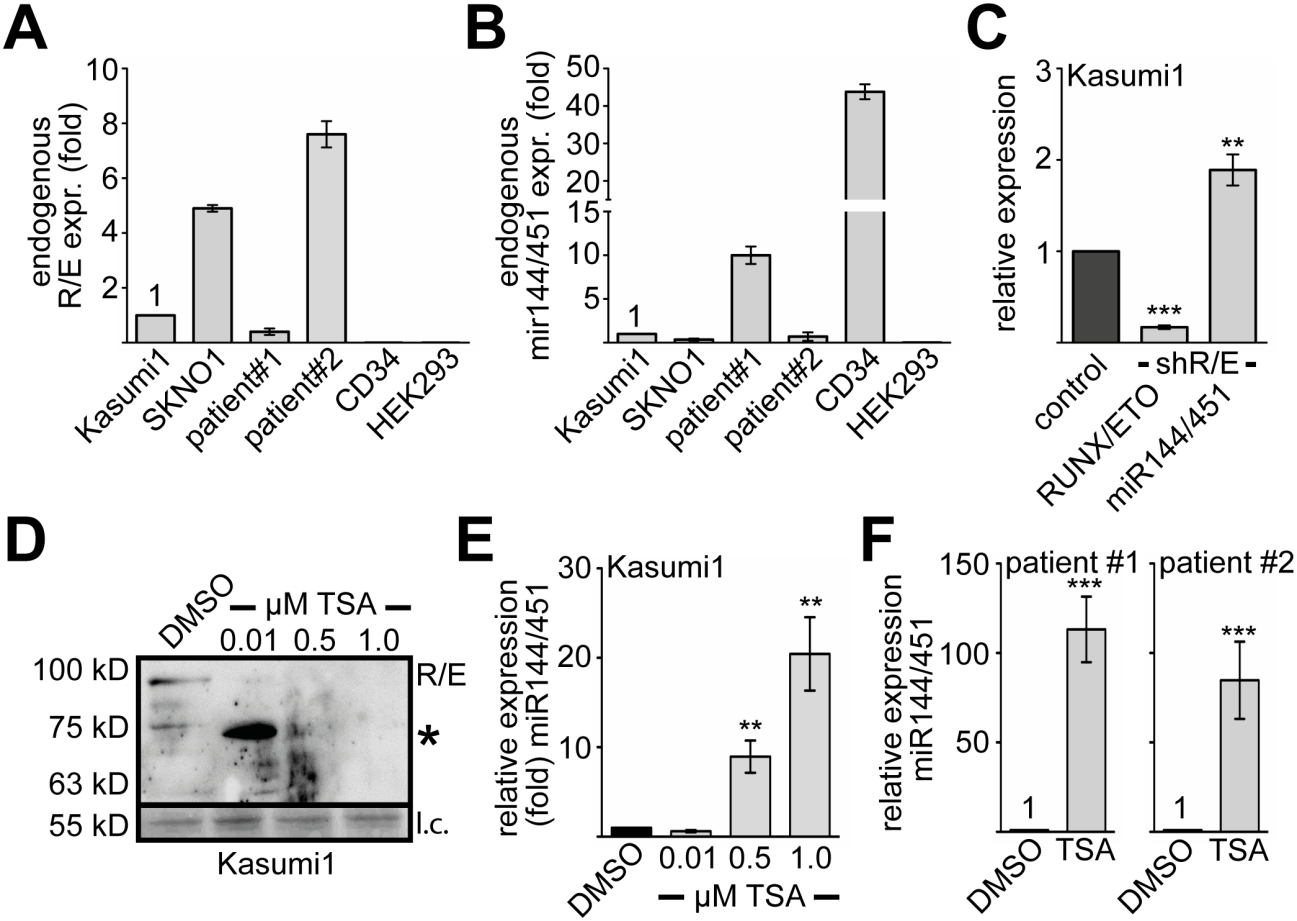
Inhibition of RUNX1/ETO increases miR144/451 expression. **(A)** Expression of endogenous RUNX1/ETO in Kasumi1, SKNO1 and two RUNX/ETO positive patient samples. Expression of RUNX1/ETO in Kasumi1 cells was set as 1 and the expression levels of the SKNO1 cells and two patient samples are shown as fold compared to Kasumi1 cells. HEK293 and hCD34+ serve as RUNX1/ETO negative controls. Values were gathered by q-RT-PCR and normalised to GAPDH. **(B)** Expression of miR144/451 in Kasumi1, SKNO1 and two different patient samples. HEK293 and hCD34+ served as miR144/451 negative and positive controls, respectively. Q-RT-PCR values were normalised to GAPDH expression and are shown as fold relative to values gathered with Kasumi1 cells. **(C)** Knock-down of RUNX1/ETO in Kasumi1 cells. Knock-down of RUNX1/ETO by an shRNA targeting the R/E fusion site leads to increased miR144/451 expression. **(D)** Treatment of Kasumi1 cells with trichostatin-A (TSA) leads to degradation of RUNX1/ETO. Kasumi1 cells were treated with the indicated concentrations of TSA for 24 hours. Protein expression was determined by Western blot using an anti-ETO antibody. R/E runs at about 100 kD. As loading control (l.c.) a protein band running at 55 kD visible upon Ponceau S staining of the membrane is shown. **(E)** MiR144/451 expression is up-regulated in Kasumi1 cells upon TSA treatment. MiR144/451 expression upon treatment with TSA was measured by q-RT-PCR. Values were normalised to GAPDH expression. **(F)** MiR144/451 expression is up-regulated in patient samples upon TSA treatment. Cells were treated with 1 uM TSA for 24 hours. Q-RT-PCR values were normalised to GAPDH expression. Q-RT-PCR against RUNX/ETO was performed with specific primers detecting the fusion protein. Error bars give the standard deviation from at least four independent determinations. The P-values were calculated using Student’s t test. **P <0.01, ***P <0.001.

Because RUNX1/ETO influences miR144/451 expression in transduced K562 and hCD34+ cells, we analysed miR144/451 expression in two RUNX1/ETO dependent cell lines (Kasumi1, SKNO1) and RUNX1/ETO positive primary AML samples (Fig 6A and 6B). RUNX1/ETO expression of Kasumi1, which is an established RUNX1/ETO model cell line, was set as one. We measured 5-fold higher RUNX1/ETO expression in SKNO1 cells, the patient samples #1 expressed 3-times less RUNX1/ETO than Kasumi1 cells and patient sample #2 expressed the highest R/E mRNA level (Fig 6A). Human primary CD34+ and HEK293 cells expressed no RUNX1/ETO (Fig 6A). Subsequently, we determined miR144/451 expression levels. Compared to normal hCD34+ cells, RUNX1/ETO expressing samples exhibited lower levels of miR144/451 (Fig 6B). To determine if endogenous RUNX1/ETO contributes to the repression of miR144/451 we knocked-down RUNX1/ETO in Kasumi1 cells as described (Fig 6C) [53]. This indeed led to increased expression of miR144/451 (Fig 6C). As it was demonstrated that the HDAC inhibitor trichostatin-A (TSA) leads to degradation of RUNX1/ETO [55], we treated Kasumi1 cells (Fig 6D) with TSA. We found that treatment of Kasumi1 cells with 0.01 uM TSA lead to degradation of RUNX1/ETO and the appearance of a degradation band, at higher TSA levels the RUNX1/ETO protein entirely disappeared (Fig 6D). When we measured miR144/451 levels in TSA treated Kasumi1 cells, we found that miR144/451 levels increased upon treatment (Fig 6E). Similarly, treatment of the primary RUNX1/ETO positive patient samples with TSA led to increase of miR144/451 expression (Fig 6F). These data strengthen the notion of a link between RUNX1/ETO and miR144/451 expression, which is sensitive to pharmacological inhibition of RUNX1/ETO.

### RUNX1/ETO and miR451 target gene expression is connected

**Fig 7:**
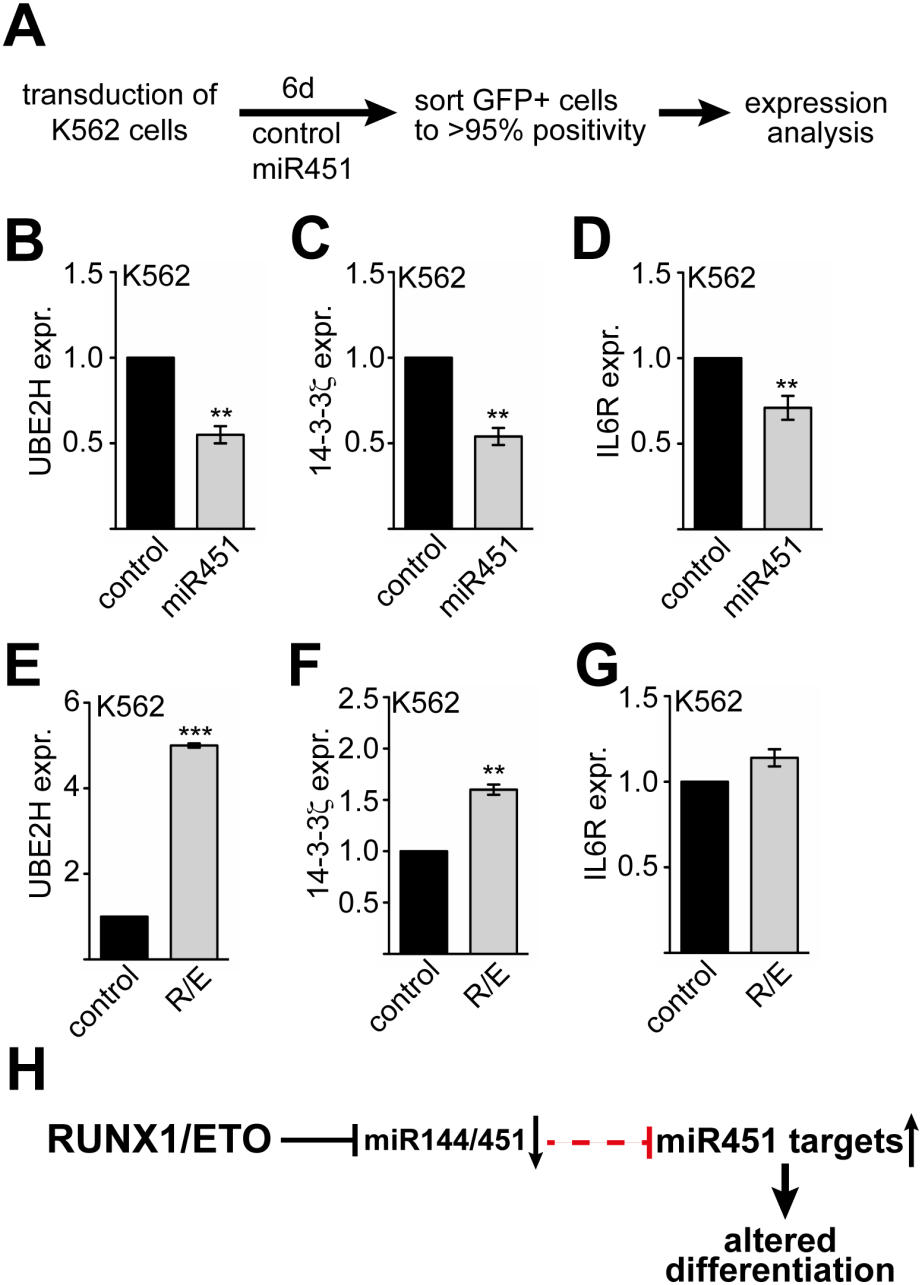
MiR451 and RUNX1/ETO influence miR451 target genes. **(A)** Schematic overview of K562 transduction with a miR451 expression vector, sorting and treatment. K562 cells were transduced, cultured for 6 days and sorted according to the GFP signal. GFP-positive cells were further analysed. **(B)** MiR451 over-expression in K562 cells leads to down-regulation of the miR451 target UBE2H at the mRNA level. **(C)** MiR451 over-expression in K562 cells leads to down-regulation of the miR451 target 14-3-3ξ at the mRNA level. **(D)** MiR451 over-expression in K562 cells leads to down-regulation of the miR451 target IL6R at the mRNA level. **(E)** Over-expression of RUNX1/ETO increases UBE2H expression at the mRNA level. **(F)** Over-expression of RUNX1/ETO increases 14-3-3ξ expression at the mRNA level. **(G)** Over-expression of RUNX1/ETO has only a minor influence on IL6R mRNA expression. **(B-G)** Q-RT-PCR values are shown as relative expression compared to K562 cells transduced with an empty control vector and normalised to GAPDH expression. The error bars represent the standard deviation from at least four independent determinations. The P-values were calculated using Student’s t test. **P< 0.01, ***P< 0.001. **(H)** Schematic representation of RUNX1/ETO activity on miR451 target genes. RUNX1/ETO represses transcription of the miR144/451 pri-microRNA, leading to less miR451 in the cells. As a consequence miR451 targets are up-regulated, which contributes to altered differentiation.

Our results suggest a connection between RUNX1/ETO expression and disturbed erythroid differentiation. As we found that mostly miR451 (and not miR144) acts on erythropoiesis (Fig 4), we were interested if RUNX1/ETO would influence the expression of miR451 targets. We over-expressed miR451 in K562 cells (Fig 7A) and analysed the expression of the known miR451 targets UBE2H, 14-3-3 and IL6R [25,27,56,57]. As microRNAs can act on mRNA stability and translation we examined the mRNA level (Fig 7) and the protein amount (S9 Fig). UBE2H, 14-3-3ξ and IL6R mRNA was decreased upon miR451 over-expression (Fig 7B–7D). Furthermore, we detected a reduction of UBE2H and 14-3-3ξ at the protein level, whereas IL6R remained unchanged (S9 Fig). In contrast, RUNX1/ETO increased UBE2H and 14-3-3ξ mRNA expression (Fig 7E and 7F), however RUNX1/ETO did not influence IL6R significantly (Fig 7G). Taken together, we detected an effect of miR451 on reported target mRNAs and could also show that RUNX1/ETO influences expression of the miR451 targets UBE2H and 14-3-3ξ. Thus, we propose that RUNX1/ETO represses expression of the miR144/451 cluster, which leads to up-regulation of miR451 targets, contributing to altered differentiation (Fig 7H).

## Discussion

Our study shows that RUNX1 influences the expression of specific microRNAs involved in megakaryocytic/erythroid differentiation. Interestingly, RUNX1 acts as an activator of some microRNAs, which suppress erythropoiesis. Thus inversely, the down-regulation of RUNX1 during erythropoiesis would diminish expression of these microRNAs and further support erythropoiesis. For example the microRNAs miR221 and miR222 are negatively influencing erythropoiesis through targeting the c-kit receptor and are both down-regulated during erythroid differentiation [58]. Similarly, down-regulation of miR223 enhances erythropoiesis through de-repressed expression of its target LMO2 [59]. MiR221/222 and miR223 are directly repressed by RUNX1/ETO [42,43], which provides a good example of the intricate relationship between RUNX1, RUNX1/ETO and microRNAs in normal differentiation and leukemia. Another group of microRNAs, which we found up-regulated by RUNX1 are known to positively influence megakaryopoiesis, such as miR34a, miR146 and miR150 [60–65]. Furthermore, we could reproduce the previously described up-regulation of miR27a by RUNX1, which was itself shown to target RUNX1, thereby implementing a feed back regulatory loop [40]. Our study also revealed that RUNX1 represses the expression of two microRNAs namely miR126 and the microRNAs of the miR144/451 cluster. MiR126 inhibits erythropoiesis from embryonic stem cells, partly by targeting the protein tyrosine phosphatase, nonreceptor type 9 (PTPN9) [66]. Interestingly, alteration of miR126 expression promotes leukemogenesis in cooperation with RUNX1/ETO [67]. Our observation that RUNX1 influences miR126 suggests a relationship between RUNX1 and RUNX1/ETO in the regulation of miR126. However, it is not known if miR126 is directly influenced by RUNX1 or RUNX1/ETO. Recently, miR126 was demonstrated to regulate self-renewal and quiescence of normal hematopoietic stem cells (HSC) and acute myeloid leukemia stem cells (LSC) [68,69]. In the same landmark paper the authors found that miR451 expression is high in the non-LSC population [68]. Whether the lower amount of miR451 in LSCs compared to HSCs is functionally significant currently remains elusive.

Because of its role in erythropoiesis we focused on miR144/451 [26,27,29,35], as we recently reported that RUNX1 epigenetically represses the key erythroid transcription factor KLF1 [5]. In line with the notion that RUNX1 inhibits the erythroid gene expression program during megakaryopoiesis, our data reveal that RUNX1 is a repressor of the erythroid miR144/451 microRNA cluster. We found that RUNX1 and TAL1 are bound to miR144/451 regulatory sequences in undifferentiated hCD34+ cells, which allows some degree of expression. However, upon megakaryocytic differentiation RUNX1 binding is increased, concomitantly RUNX1 associated corepressors such as PRMT6 (protein arginine methyltransferase 6) [6,7] are recruited. These trigger repressive histone modification marks and miR144/451 is down-regulated. Repression of miR144/451 expression by RUNX1 is in line with the observation that expression of miR144/451 increases during erythroid differentiation, when RUNX1 expression is down-regulated [8,9]. In contrast miR144/451 expression is positively regulated by the transcription factors TAL1 and GATA1. Both transcription factors have been shown to be important activator of the erythroid gene expression program [70–74]. Taken together our data confirm that miR144/451 is an erythroid microRNA [26,35,37], which is activated by TAL1/GATA1 but repressed by RUNX1 during megakaryopoiesis.

RUNX1 has been mainly described as a transcriptional activator, however it can also act as a transcriptional repressor [5,6,75–79]. In contrast, RUNX1/ETO mostly acts as a transcriptional repressor, which recruits a corepressor complex including N-CoR and Sin3 [11,13,49] although in some cases RUNX1/ETO also activates genes [80]. Because RUNX1 and RUNX1/ETO are simultaneously expressed in leukemic cells it is an interesting question how they interact on miR144/451 expression. Genome wide studies have shown that RUNX1/ETO and RUNX1 compete for binding to the same binding sites [24] and it has been suggested that RUNX1/ETO serves as a dominant negative inhibitor of RUNX1 function [49]. However recent data imply that RUNX1/ETO does not act exclusively as a dominant negative repressor of RUNX1 function [80]. Knock-down of RUNX1/ETO leads to genome wide changes of the chromatin structure and to novel RUNX1 binding sites at places where no RUNX1/ETO was bound before [81]. Furthermore, cell lines expressing RUNX1/ETO, such as Kasumi1, grow dependent on the presence of wild type RUNX1 [82]. At the molecular level there is evidence of a dynamic balance of RUNX1 and RUNX1/ETO activity in leukemia cells [24]. Our data show that RUNX1 and RUNX1/ETO not exclusively compete functionally, as both can act as repressor on the miR144/451 promoter. This confirms that both can have a similar function on a target gene. Our data imply that both RUNX1 and RUNX1/ETO inhibit the erythroid gene expression program ([5], this study). However, RUNX1 represses erythroid gene expression upon megakaryocytic differentiation, upon up-regulation of RUNX1 [5]. In contrast, the t(8;21) takes place in an early stem cell or progenitor stage and can even be detected in healthy newborn children [83]. Accordingly, differentiation of RUNX1/ETO cells is blocked at an early myeloid stage and about 40% of the immature M2-type of leukemia are RUNX1/ETO dependent [10]. Our data imply that RUNX1/ETO would repress expression of miR144/451 similar to RUNX1. But RUNX1/ETO would repress miR144/451 at an inappropriate differentiation stage and thus interfere with normal differentiation contributing to impaired lineage fidelity of RUNX1/ETO expressing cells.

In line with this notion the RUNX1/ETO fusion protein was found to repress erythroid differentiation [22,50–52]. This effect was dependent on the presence of the NHR4 domain within the C-terminal domain of the fusion protein [22]. However, the inhibitory effect of RUNX1/ETO was not consistent in all studies [84] and the effect of the RUNX1/ETO truncated form (REtr or RE9a) was not tested. Our results show that full length RUNX1/ETO and the truncated RUNX1/ETOtr equally decreased erythroid differentiation of primary hCD34+ cells. Notably, the repression of miR144/451 by RUNX1/ETO was released by knock-down of RUNX1/ETO in Kasumi1 cells. Furthermore, treatment of primary AML patient samples with TSA, which leads to degradation of RUNX1/ETO [55], increases miR144/451 expression. Additionally, we showed that RUNX1/ETO expression leads to an up-regulation of the miR451 targets UBE2H and 14-3-3ξ. Notably, the connection of miR451 with 14-3-3ξ expression has been linked to erythroid differentiation [27]. This supports the idea that RUNX1/ETO represses miR451 expression thereby inducing the expression of miR451 target mRNAs.

Our results and data gathered by others [23,43] suggest that RUNX1 acts as master regulator of a regulatory microRNA network in hematopoietic differentiation, which is disturbed by the leukemogenic RUNX1/ETO fusion product.

## Material and Methods

### Cell culture

K562, Kasumi1, SKNO1 and HEK293T/17 (ATCC CRL-11268) cells were cultured in Roswell Park Memorial Institute 1640 medium (RPMI; Life Technologies) and Dulbecco’s modified eagle medium (DMEM; Life Technologies), respectively. Supplements were 10% fetal calf serum (FCS), 2 mM glutamine and 1% penicillin/streptomycin. Kasumi1 cells were supplemented with 20% FCS. SKNO1 cells were supplemented with 10 ng/mL granulocyte-macrophage colony stimulation factor (GM-CSF). K562 cells were treated with 30 nM 12-o-tetradecanylphorbol-13-acetate to induce megakaryocytic differentiation. For erythroid differentiation K562 cells were incubated with 30 μM hemin. For TSA treatment three concentrations of trichostatin-A (TSA) (Sigma Aldrich), 0.01 μM, 0.5 μM and 1.0 μM were used. Cell density was set to 0,5x10^6^ cells/ml incubation with TSA was for 24 hours. Gene expression was analysed by quantitative real-time PCR. Primary AML-patient samples were gathered with written informed consent. G-CSF mobilized human primary CD34+ cells were from healthy volunteer donors with written informed consent. The local ethics committee approved the experiments (permit #329–10).

### Primary human CD34+ cell culture

hCD34+ cell were isolated from G-CSF mobilized peripheral blood using immunomagnetic selection according to the manufacturer’s instructions (Miltenyi Biotec, Bergisch Gladbach, Germany). Thereafter, the hCD34+ cells were expanded for 3 days in serum-free expansion medium (StemSpan SFEM, Stemcell Technologies, Grenoble, France) supplemented with 100 ng/ml FLT-3, 100 ng/ml SCF, 20 ng/ml IL-3 and 20 ng/ml IL-6 (Miltenyi Biotec). hCD34+ cells were transduced with lentiviral vectors expressing also green fluorescent protein (GFP), which enabled sorting the cells according to their GFP signal (FACSAria, BD-Biosciences, Heidelberg).

Transductions were performed with an MOI of 25. Knock-down constructs for shRUNX1/ETO were designed using the SEW-backbone as described [85,86]. The SiEW or the LEGO lentiviral vector was used for over-expression [48]. Transduced hCD34+ cells were either seeded out on methylcellulose plates for CFU assay according to the manufacturer’s instructions (StemMACS HSC-CFU with Epo, Miltenyi Biotec). Colonies were counted on day 12 after seeding. To induce erythroid differentiation the expanded hCD34+ cells were cultured in StemSpan serum-free expansion medium II (SFEM II, Stemcell Technologies) supplemented 20 ng/ml SCF, 5 ng/ml IL-3, 2 μM dexamethasone (Sigma-Aldrich, Munich, Germany), 0,2 μM estradiol (Sigma-Aldrich) and 1U/ml erythropoietin (PeproTech, Hamburg, Germany) [87]. For megakaryocytic differentiation hCD34+ cells were incubated with SFEM II supplemented with megakaryocyte expansion cytokine cocktail (StemSpan Megakaryocyte Expansion Supplement, Stemcell Technologies). Differentiation was verified by RT-PCR for differentiation markers [5,6] and by FACS (S7 Fig). ChIP experiments were performed with hCD34+ differentiated cells (upon 6 days) and compared to hCD34+ cells cultured in expansion medium for the same time. Experiments were performed with hCD34+ cells from at least two independent donors.

### Chromatin immunoprecipitation

Preparation of cell lysates for chromatin immunoprecipitation (ChIP) assays was performed according to the X-ChIP protocol (Abcam) with modifications [5]. 2.5–10 μg of antibodies were used for immunoprecipitation. ChIP-DNA was purified using DNA purification columns (ChIP DNA Clean and Concentrator, Zymo Research, USA) and eluted with 40 μl TE-buffer. DNA was analyzed by quantitative PCR. Antibodies and ChIP-PCR primers are given in S2 File. DNA recovery was calculated as percent of the input, bars represent the standard deviation from at least four independent determinations. ChIP-values of histone modification were corrected for nucleosome density using values gathered by a Histone 3 ChIP.

### Luciferase assay

The human miR144/451 promoter region (-750 bp) was cloned into the pGL4.10 vector. The enhancer region (3410–4680 bp) of miR144/451 was cloned in front of a minimal promoter into the pGL4.23 vector. K562 and HEK293T cells were transfected with Metafectene (Biontex, Martinsried/Planegg, Germany) with the reporter plasmids and a β-galactosidase expressing vector. 48 h after transfection the luciferase and β-galactosidase activity was analyzed. To control for transfection efficiency the firefly luciferase activity was normalized to β-galactosidase.

### RNA isolation and quantitative RT-PCR

For RNA isolation the miRNeasy Mini Kit (Qiagen, Valencia, CA, USA) was used to purify a miRNA-enriched fraction and a total RNA fraction separately. cDNA was synthesized from the total RNA fraction using Omniscript reverse transcriptase kit (Qiagen). Quantitative RT-PCR was performed using a LightCycler 480 (Roche, Mannheim, Germany) and SYBR-Green PCR MasterMix (Eurogentec, Köln, Germany). Relative amounts of mRNA were calculated by the ΔΔCt method using GAPDH or TBP as controls. To detect and quantify mature miRNAs a Taqman assay (Life Technologies) or the miScript SYBR Green PCR Kit (Qiagen) was used. Primer pairs, Taqman probes and primer assays are given in supplementary material.

### Small RNA sequencing

Total RNA was isolated from K562 cells using the Trizol (Invitrogen) method according to the manufacturer's recommendations. Afterwards, the samples were DNAse I (Sigma) treated in order to remove DNA contamination. RNA quality was determined using the Agilent 2100 Bioanalyzer (Agilent Technologies, Santa Clara, CA, USA) microfluidic electrophoresis. Only samples with comparable RNA integrity numbers were selected for deep sequencing. Library preparation for small RNA-Seq was performed using the TruSeq Small RNA Sample Preparation Kit from Illumina (catalog RS-200-0012) starting from 1000 ng of total RNA. Accurate quantitation of cDNA libraries was performed using the QuantiFluorTM dsDNA System (Promega). The size range of cDNA libraries was determined applying the DNA 1000 chip on the Bioanalyzer 2100 from Agilent (140–160 bp). cDNA libraries were amplified and sequenced by using the cBot and HiSeq2000 from Illumina (SR, 1x51 bp, 4 GB per sample). Sequence images were transformed with Illumina software BaseCaller to bcl files, which were demultiplexed to fastq files with CASAVA (version 1.8.2). Quality check was done via FastQC (version 0.10.1, Babraham Bioinformatics). Differentially expressed small RNA molecules were identified using the OASIS platform with standard parameters. RNAs were included for further analysis if they displayed an at least -0.5 or +0.5 log2-fold change and a P-value <0.05. Sequencing data are available at the GEO-database under the accession number GSE70942.

## Supporting Information

S1 Fig Expression changes of microRNAs upon RUNX1 over expression, involved at the MEP branching. The log2 fold changes according to the sequencing date are shown and the fold changes according to q-RT-PCR detecting the mature microRNAs (values as in Fig 1E).
(TIF)

S2 Fig Regulation of miR144/451 expression. **(A-C) MiR144/451 is regulated by RUNX1.** (A) RUNX1 over-expression leads to a decrease of miR144/451 transcript in K562 cells (**same graph as in Fig 1F**). (B) RUNX1 knock-down increases miR144/451 amount in K562 cells. (C) Western blot showing RUNX1 over-expression and RUNX1 knock-down, respectively. (D-F) MiR144/451 expression is regulated by TAL1. (D) TAL1 over-expression slightly increases miR144/451 expression in K562 cells. (E) Knock-down of TAL1 decreases miR144/451 expression in K562 cells. (F) Western blot showing TAL1 over-expression and TAL1 knock-down in K562 cells, respectively. (G-I) GATA1 regulates miR144/451 expression. (G) GATA1 over-expression increases miR144/451 expression in K562 cells. (H) GATA1 knock-down decreases miR144/451 expression in K562 cells. (I) Western blot showing GATA1 over-expression and GATA1 knock-down in K562 cells, respectively. Q-RT-PCR was performed with specific primers for the given mRNAs. Error bars give the standard deviation of at least four independent determinations. P-values were calculated by Student’s t test. *P <0.05, **P <0.01, ***P <0.001.
(TIF)

S3 Fig Genomic region 5‘ of the miR144/451 cluster. Screenshot from the human genome browser: Shown is the genomic region of miR144/451. The coding regions of miR144 and miR451 are marked by red boxes. These regions display a high degree of conservation. Two regions 5‘ of the coding region display a higher degree of conservation and are marked with a green bar (Enhancer Region) and a blue bar (Promoter Region). These regions have heightened H3K27Ac marks, which is indicative of regulatory regions (blue). In addition the enhancer region and the promoter region have a high density of transcription factor binding, which is indicated by the black bar within the figure (Txn Factor ChIP).
(TIF)

S4 Fig **(A) ChIP analysis shows binding of TAL1 to the enhancer and promoter regions of miR144/451 in K562 cells.** (B-F) ChIP analysis shows alterations at the miR144/451 promoter upon megakaryocytic differentiation of K562 cells (K562-M). (B) Binding of TAL1 to the promoter region of miR144/451 before and after megakaryocytic differentiation. (C-D) H3R2me and the corresponding PRMT6 at the promoter region of miR144/451 before and after megakaryocytic differentiation. (F-G) WDR5 and the corresponding H3K4me3 at the miR144/451 promoter before and after megakaryocytic differentiation. Q-PCR was performed with primers binding in the promoter region of miR144/451. Values are given as percent input. Histone modification ChIPs were normalised to values gathered with an anti Histone3 ChIP. Error bars represent the deviation from at least four independent experiments. P-values were calculated according to Student’s test. **P <0.01, ***P <0.001.
(TIF)

S5 Fig **(A) MiR144/451 expression is decreased upon megakaryocytic differentiation and increased upon erythroid expression of K562 cells.** Expression of the endogenous pri-microRNA was measured by q-RT-PCR. (B) Analysis of RUNX1 protein abundance in K562 cells and upon megakaryocytic differentiation (K562-M) by Western blot using anti-RUNX1 antibody and anti-Lamin antibody as control. (C) ChIP assay analysis of RUNX1 binding to the miR144/451 promoter during erythroid (K562-E) or megakaryocytic (K562-M) differentiation of K562 cells.
(TIF)

S6 Fig The mature microRNAs miR144 and miR451 increase upon erythroid differentiation to a different degree. hCD34+ cell were differentiated towards the erythroid linage for 6 days. The mature micro RNAs were determined by q-RT-PCR values were normalised to RNU6-2 expression and are shown as fold over values gathered for undifferentiated hCD34+ cells. Error bars give the standard deviation of at least four independent determinations. P-values were calculated using Student’s t test. *P < 0.05. **P < 0.01.
(TIF)

S7 Fig **(A) Scheme of hCD34+ CFU assay upon transduction.** Upon isolation hCD34+ cells were expanded for 3 days and transduced. After two days the cells were sorted and GFP positive cells were seeded in 3.5 cm dishes. (B) Erythroid differentiation of hCD34+ cells. Cells were expanded for 3 days and subjected to erythroid differentiation. The cells erythroid cell surface marker GYPA and CD71 increase as shown by FACS. (C) Megakaryocytic differentiation of hCD34+ cells. Cells were expanded for 3 days and megakaryocytic differentiation was induced. Shown is the increase of the megakaryocytic surface marker CD61 as measured by FACS.
(TIF)

S8 Fig **(A) Screen shot of the genomic region of miR144/451 (altered).** The miR144 and miR451 coding regions are highly conserved. The genomic region, which was cloned into a lentiviral expression vector (compare Fig 4) is marked. (B) Expression validation of the miR144/451 constructK562 cells were transfected with the miR144/451 pri-microRNA expression vector. Expression of the mature microRNAs miR144 and miR451 was determined using Taq-man q-RT-PCR. (C) The cloned miR144/451 genomic region is given. The miR144 region is marked in bold, the miR451 region is marked in red. The micro-RNA coding regions, which were mutated are underlined (compare Fig 4). The grey areas are the primer regions used for cloning.
(TIF)

S9 Fig Influence of miR451 over-expression on its targets UBE2H, 14-3-3z and IL6R. The effect of miR451 over-expression on protein levels was determined in K562 cells. Western blot was performed using specific antibodies against the given proteins. Tubulin was used as loading control.
(TIF)

S1 File Supplementary data file. (XLSX)

S2 File Supplementary material. (DOCX)
