# Supplementary figures and images for: MiR144/451 Expression Is Repressed by RUNX1 During Megakaryopoiesis and Disturbed by RUNX1/ETO
Source: PLoS Genet. 2016 Mar 18;12(3):e1005946. doi: 10.1371/journal.pgen.1005946 (PMC4798443; doi:10.1371/journal.pgen.1005946)

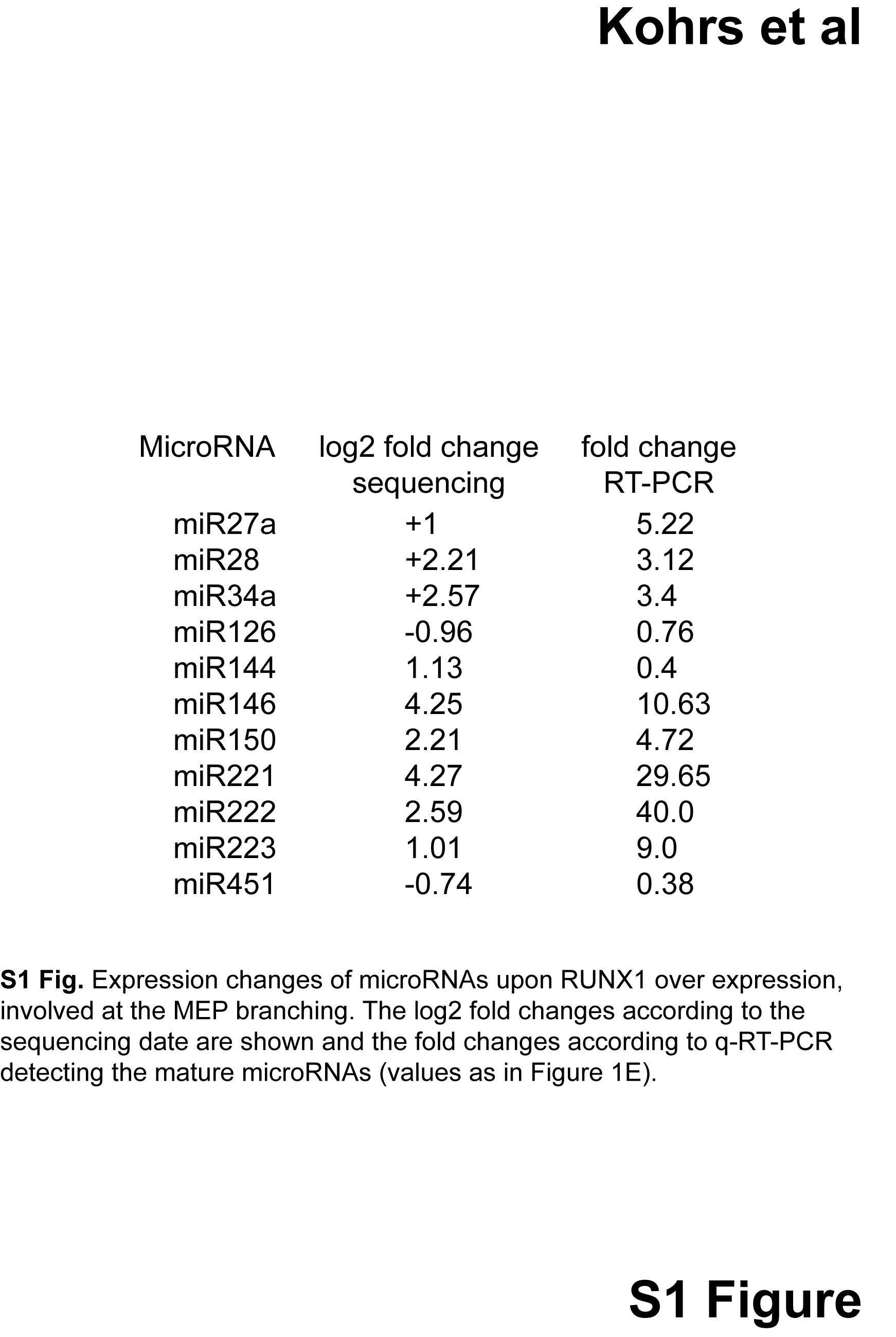

Supplement: S1 Fig — The log2 fold changes according to the sequencing date are shown and the fold changes according to q-RT-PCR detecting the mature microRNAs (values as in Fig 1E). (TIF) [file pgen.1005946.s001.tif]

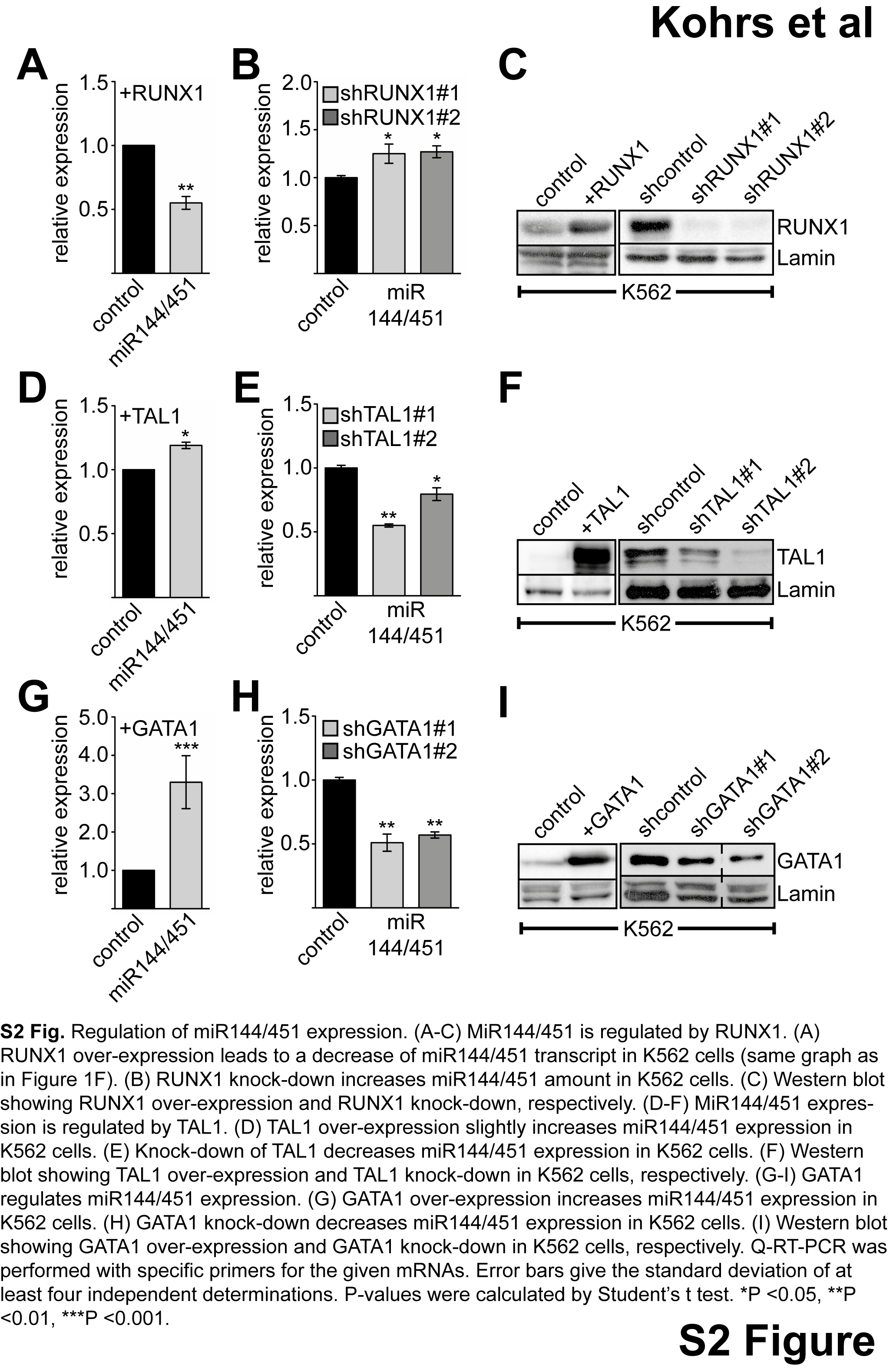

Supplement: S2 Fig — (A-C) MiR144/451 is regulated by RUNX1. (A) RUNX1 over-expression leads to a decrease of miR144/451 transcript in K562 cells (same graph as in Fig 1F). (B) RUNX1 knock-down increases miR144/451 amount in K562 cells. (C) Western blot showing RUNX1 over-expression and RUNX1 knock-down, respectively. (D-F) MiR144/451 expression is regulated by TAL1. (D) TAL1 over-expression slightly increases miR144/451 expression in K562 cells. (E) Knock-down of TAL1 decreases miR144/451 expression in K562 cells. (F) Western blot showing TAL1 over-expression and TAL1 knock-down in K562 cells, respectively. (G-I) GATA1 regulates miR144/451 expression. (G) GATA1 over-expression increases miR144/451 expression in K562 cells. (H) GATA1 knock-down decreases miR144/451 expression in K562 cells. (I) Western blot showing GATA1 over-expression and GATA1 knock-down in K562 cells, respectively. Q-RT-PCR was performed with specific primers for the given mRNAs. Error bars give the standard deviation of at least four independent determinations. P-values were calculated by Student’s t test. *P <0.05, **P <0.01, ***P <0.001. (TIF) [file pgen.1005946.s002.tif]

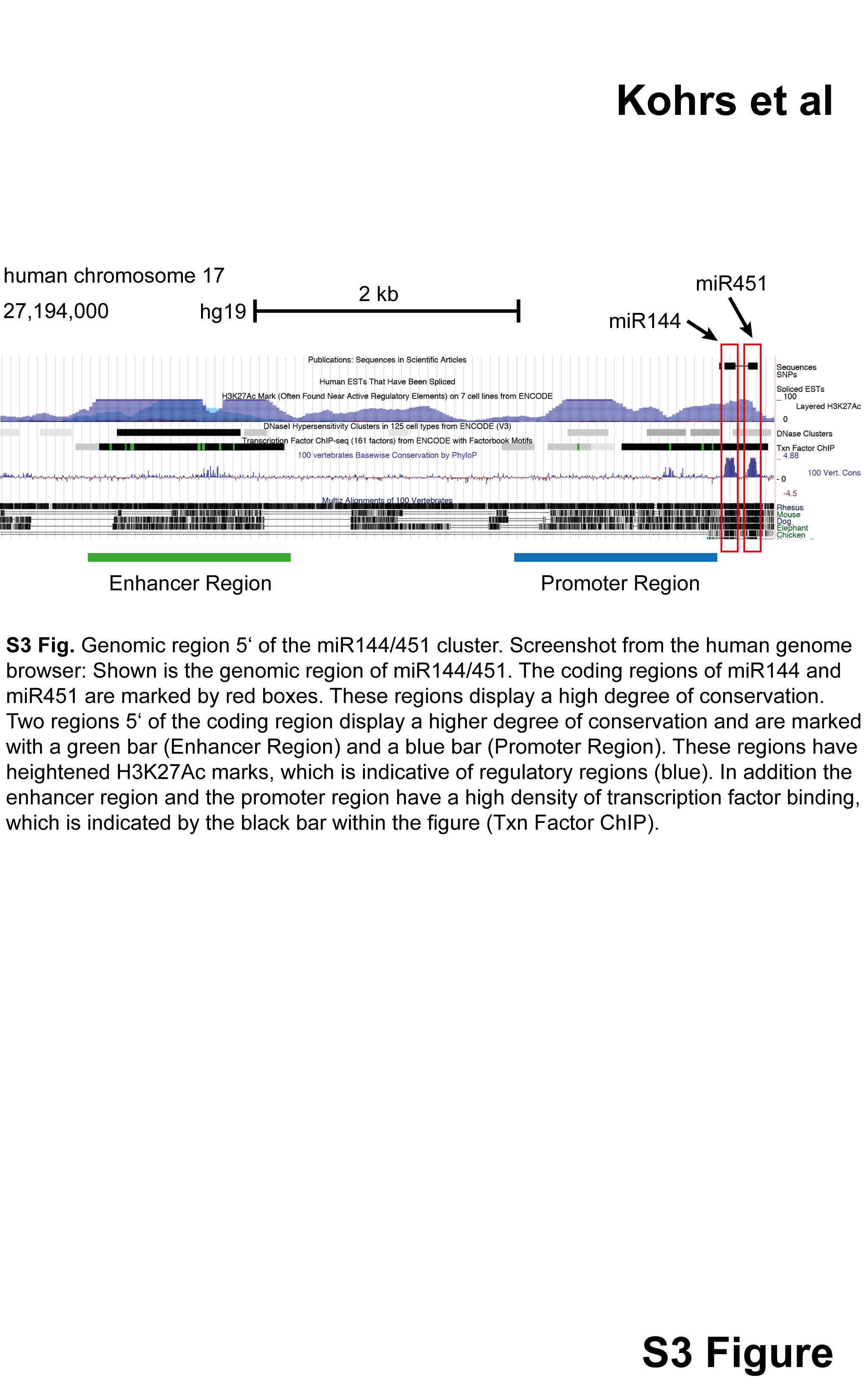

Supplement: S3 Fig — Screenshot from the human genome browser: Shown is the genomic region of miR144/451. The coding regions of miR144 and miR451 are marked by red boxes. These regions display a high degree of conservation. Two regions 5‘ of the coding region display a higher degree of conservation and are marked with a green bar (Enhancer Region) and a blue bar (Promoter Region). These regions have heightened H3K27Ac marks, which is indicative of regulatory regions (blue). In addition the enhancer region and the promoter region have a high density of transcription factor binding, which is indicated by the black bar within the figure (Txn Factor ChIP). (TIF) [file pgen.1005946.s003.tif]

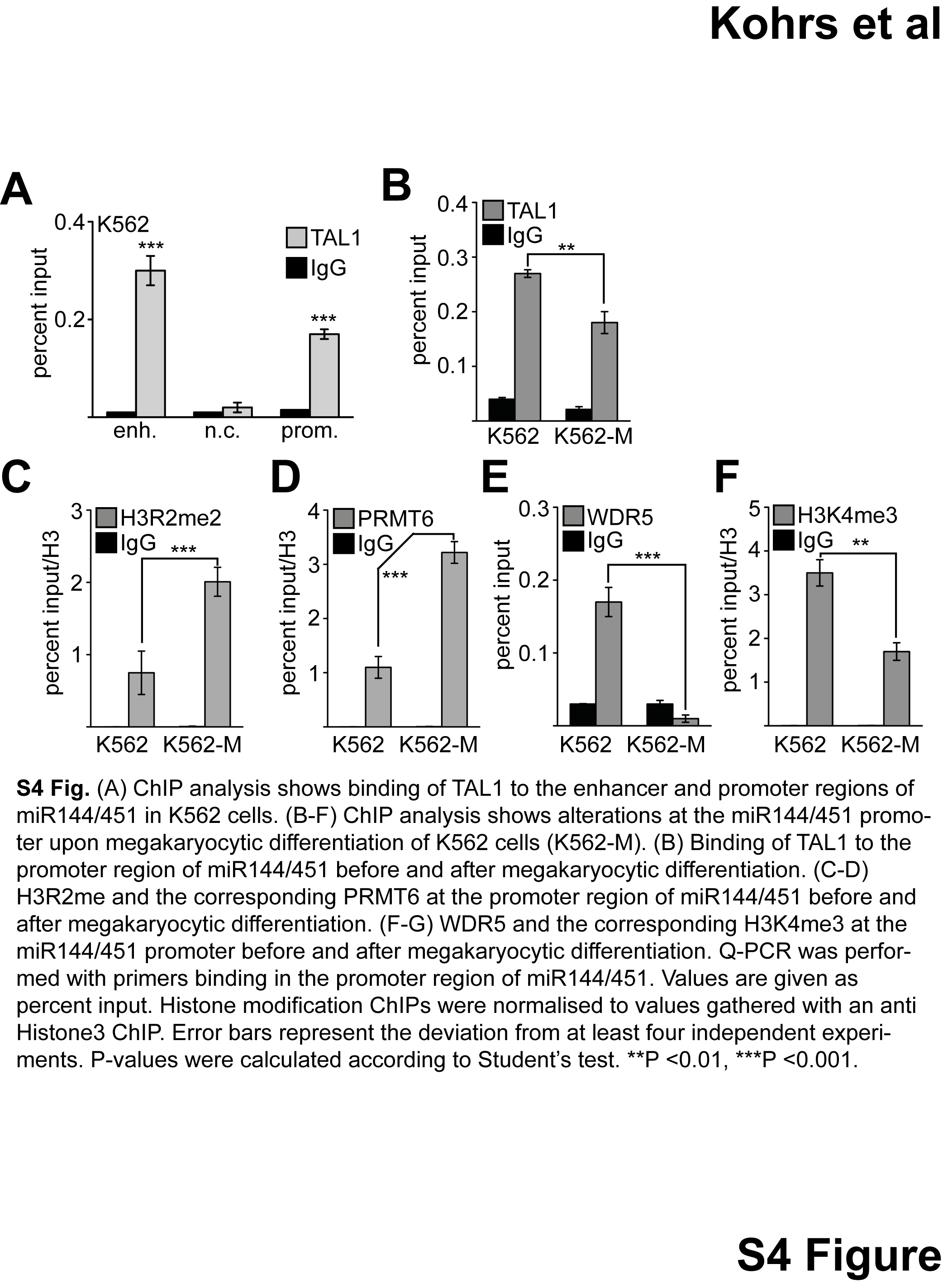

Supplement: S4 Fig — (A) ChIP analysis shows binding of TAL1 to the enhancer and promoter regions of miR144/451 in K562 cells. (B-F) ChIP analysis shows alterations at the miR144/451 promoter upon megakaryocytic differentiation of K562 cells (K562-M). (B) Binding of TAL1 to the promoter region of miR144/451 before and after megakaryocytic differentiation. (C-D) H3R2me and the corresponding PRMT6 at the promoter region of miR144/451 before and after megakaryocytic differentiation. (F-G) WDR5 and the corresponding H3K4me3 at the miR144/451 promoter before and after megakaryocytic differentiation. Q-PCR was performed with primers binding in the promoter region of miR144/451. Values are given as percent input. Histone modification ChIPs were normalised to values gathered with an anti Histone3 ChIP. Error bars represent the deviation from at least four independent experiments. P-values were calculated according to Student’s test. **P <0.01, ***P <0.001. (TIF) [file pgen.1005946.s004.tif]

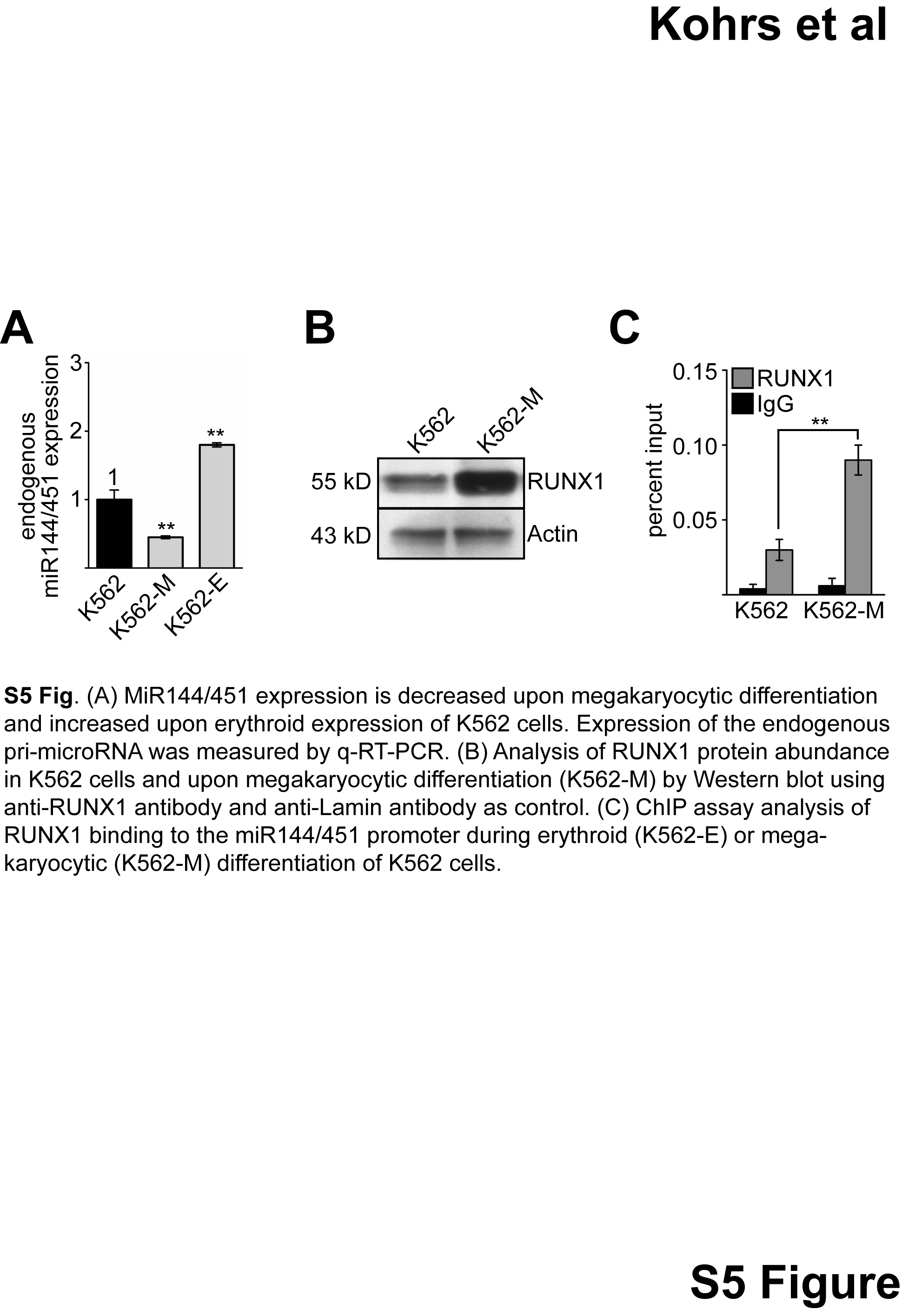

Supplement: S5 Fig — (A) MiR144/451 expression is decreased upon megakaryocytic differentiation and increased upon erythroid expression of K562 cells. Expression of the endogenous pri-microRNA was measured by q-RT-PCR. (B) Analysis of RUNX1 protein abundance in K562 cells and upon megakaryocytic differentiation (K562-M) by Western blot using anti-RUNX1 antibody and anti-Lamin antibody as control. (C) ChIP assay analysis of RUNX1 binding to the miR144/451 promoter during erythroid (K562-E) or megakaryocytic (K562-M) differentiation of K562 cells. (TIF) [file pgen.1005946.s005.tif]

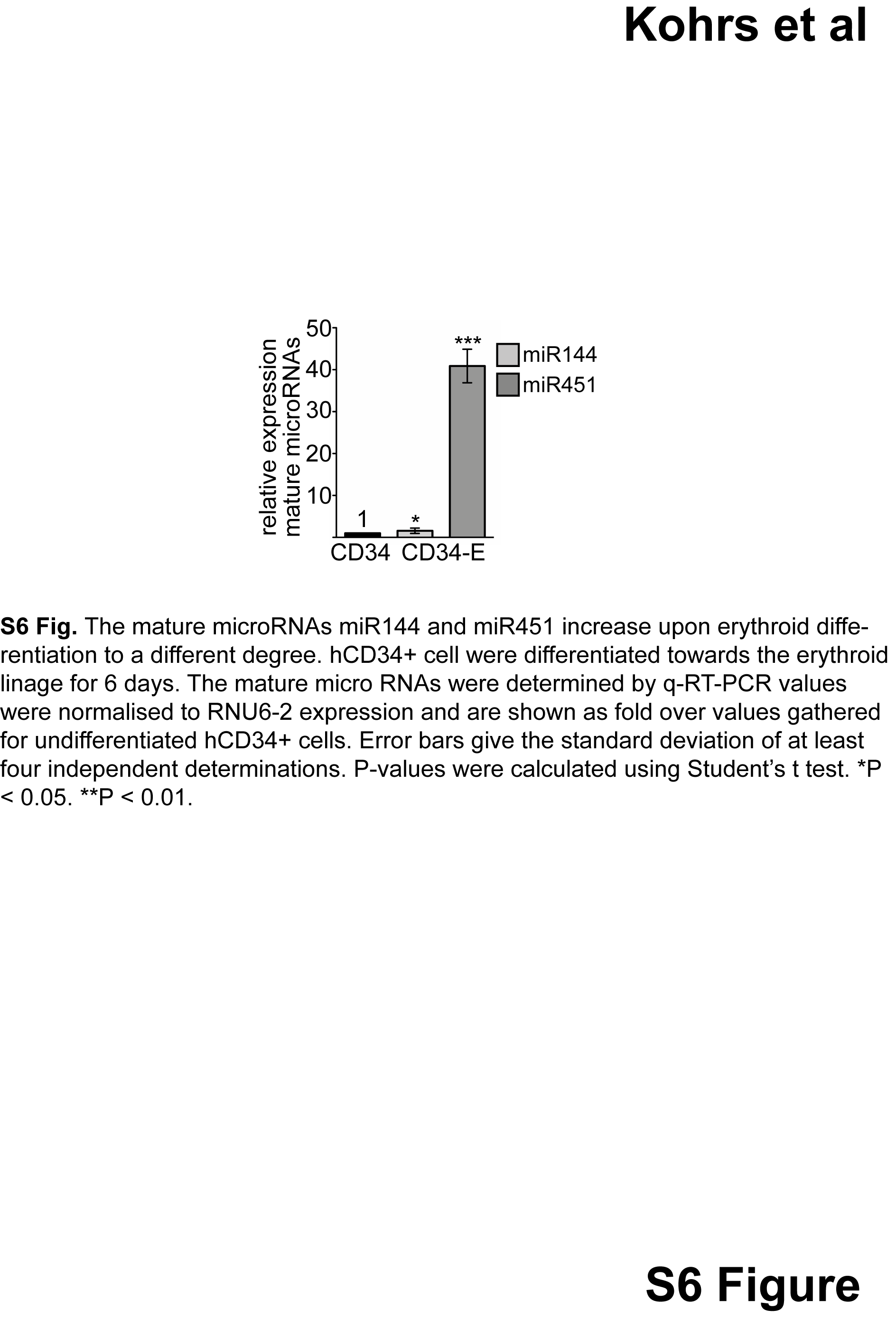

Supplement: S6 Fig — hCD34+ cell were differentiated towards the erythroid linage for 6 days. The mature micro RNAs were determined by q-RT-PCR values were normalised to RNU6-2 expression and are shown as fold over values gathered for undifferentiated hCD34+ cells. Error bars give the standard deviation of at least four independent determinations. P-values were calculated using Student’s t test. *P < 0.05. **P < 0.01. (TIF) [file pgen.1005946.s006.tif]

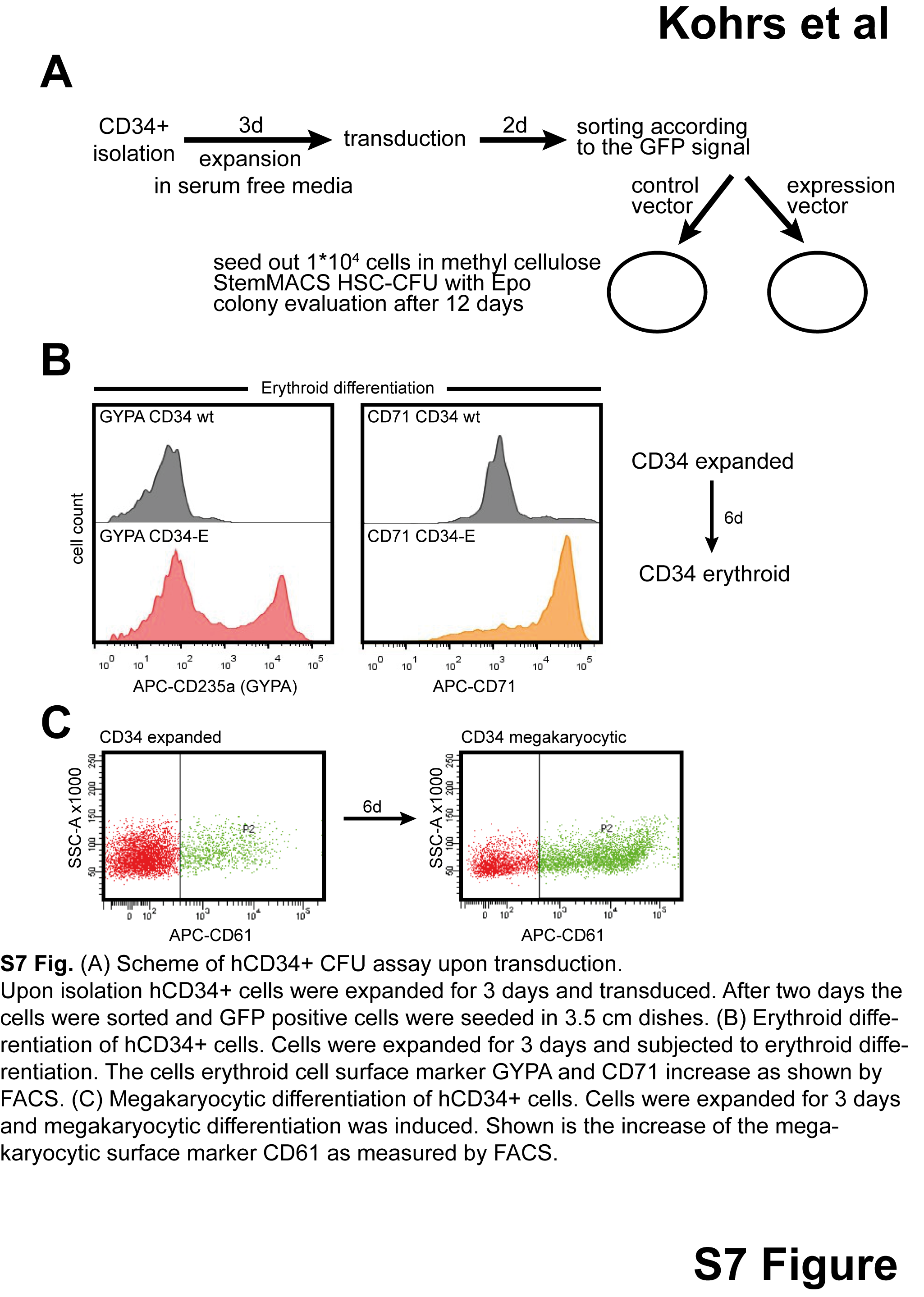

Supplement: S7 Fig — (A) Scheme of hCD34+ CFU assay upon transduction. Upon isolation hCD34+ cells were expanded for 3 days and transduced. After two days the cells were sorted and GFP positive cells were seeded in 3.5 cm dishes. (B) Erythroid differentiation of hCD34+ cells. Cells were expanded for 3 days and subjected to erythroid differentiation. The cells erythroid cell surface marker GYPA and CD71 increase as shown by FACS. (C) Megakaryocytic differentiation of hCD34+ cells. Cells were expanded for 3 days and megakaryocytic differentiation was induced. Shown is the increase of the megakaryocytic surface marker CD61 as measured by FACS. (TIF) [file pgen.1005946.s007.tif]

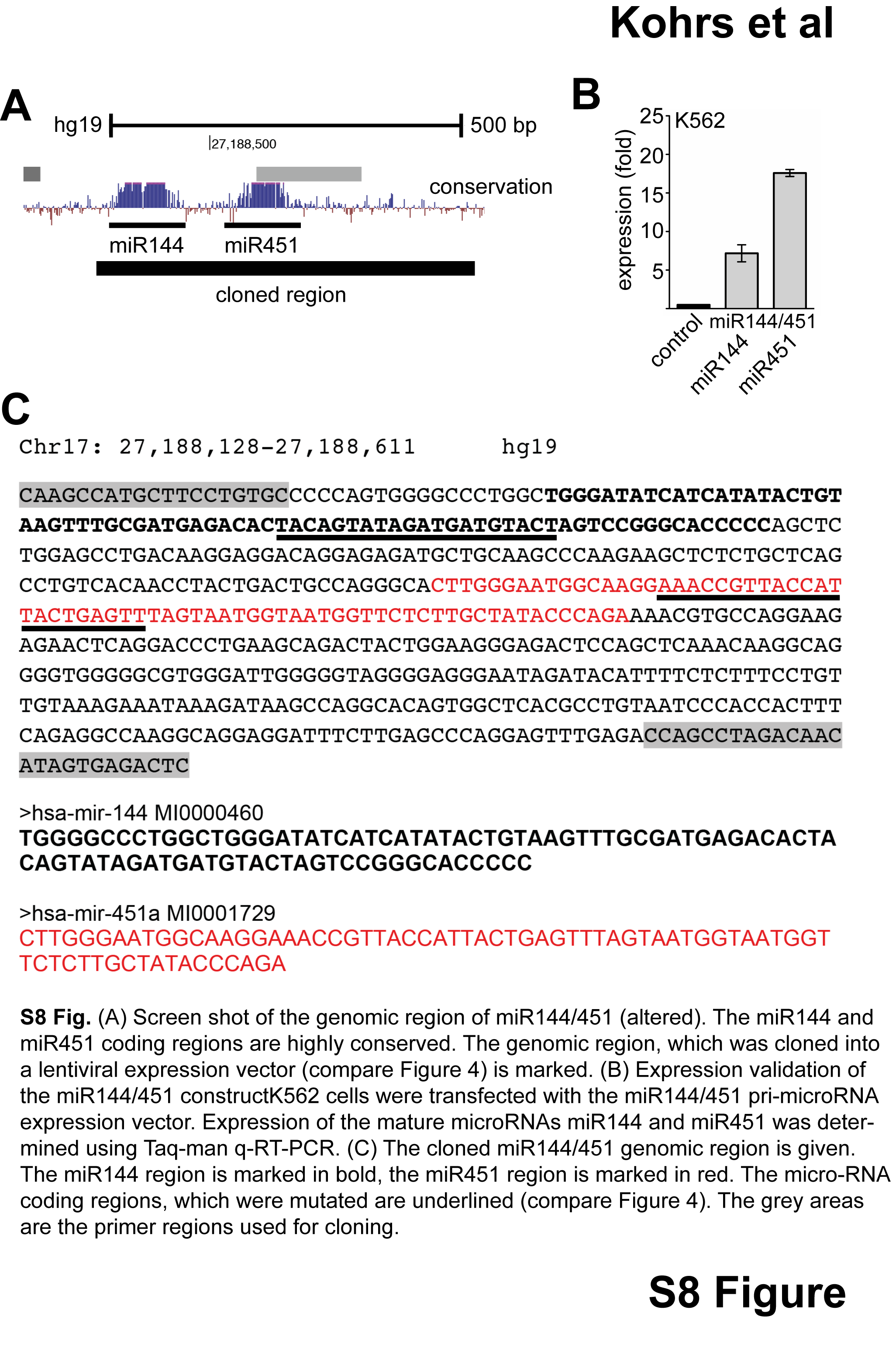

Supplement: S8 Fig — (A) Screen shot of the genomic region of miR144/451 (altered). The miR144 and miR451 coding regions are highly conserved. The genomic region, which was cloned into a lentiviral expression vector (compare Fig 4) is marked. (B) Expression validation of the miR144/451 constructK562 cells were transfected with the miR144/451 pri-microRNA expression vector. Expression of the mature microRNAs miR144 and miR451 was determined using Taq-man q-RT-PCR. (C) The cloned miR144/451 genomic region is given. The miR144 region is marked in bold, the miR451 region is marked in red. The micro-RNA coding regions, which were mutated are underlined (compare Fig 4). The grey areas are the primer regions used for cloning. (TIF) [file pgen.1005946.s008.tif]

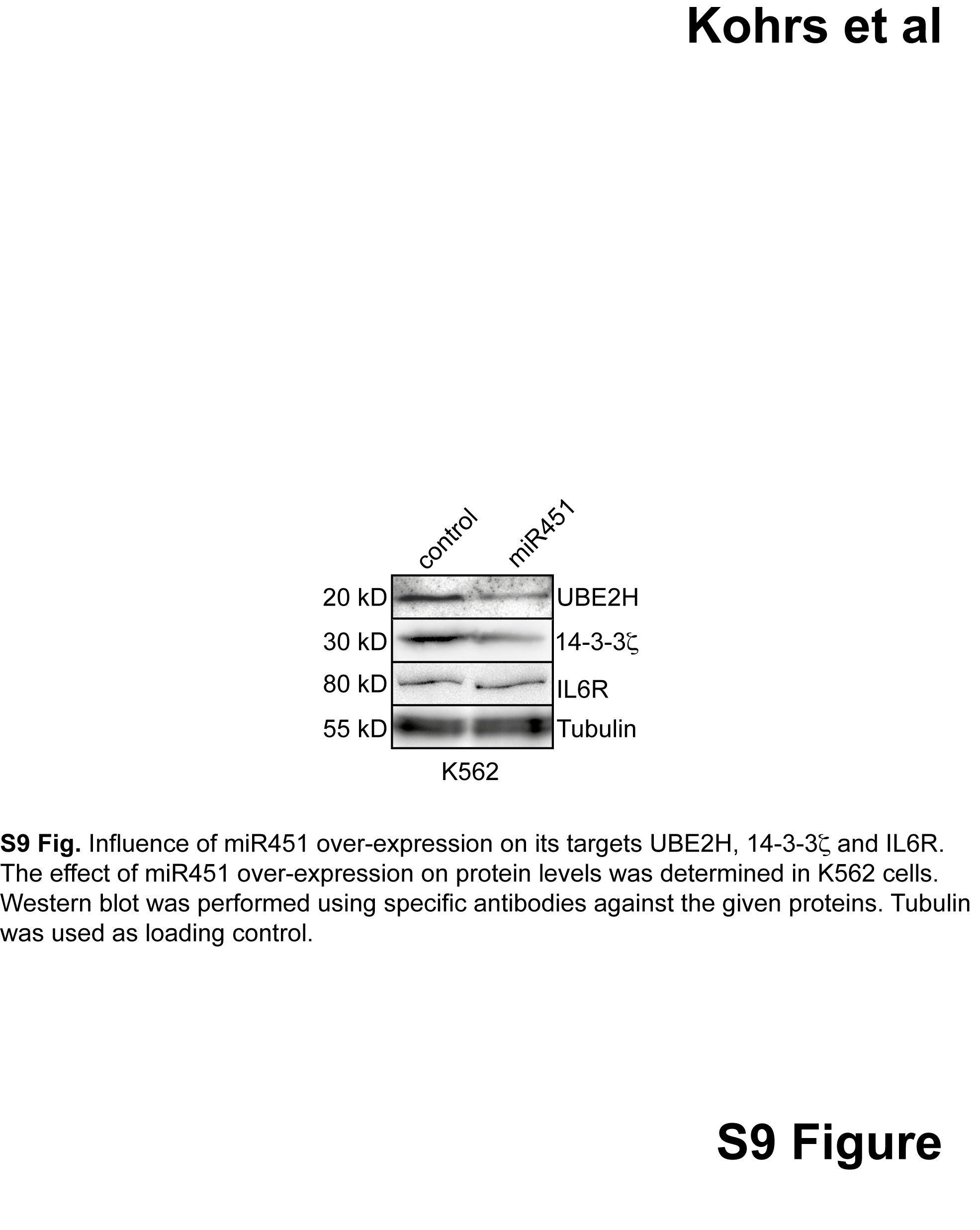

Supplement: S9 Fig — The effect of miR451 over-expression on protein levels was determined in K562 cells. Western blot was performed using specific antibodies against the given proteins. Tubulin was used as loading control. (TIF) [file pgen.1005946.s009.tif]
