# Supplementary material for: MiR144/451 Expression Is Repressed by RUNX1 During Megakaryopoiesis and Disturbed by RUNX1/ETO
Source: PLoS Genet. 2016 Mar 18;12(3):e1005946. doi: 10.1371/journal.pgen.1005946 (PMC4798443; doi:10.1371/journal.pgen.1005946)
Supplement: S2 File — (DOCX) [file pgen.1005946.s011.docx]

**Supplementary Material**

**Antibodies used for ChIP**

H3K4me3 (ab1012, Abcam), TAL1 (ab75739, Abcam), ETO (sc-9737, Santa Cruz), HA-tag (ab9110, Abcam), RNApol II (ab5408, Abcam), RUNX1 (ab23980, Abcam), PRMT6 (sc-55702, Santa Cruz), p300 (MS-586, NeoMarkers), WDR5 (sc-100895, Santa Cruz), H3R2me2 (ab80075, Abcam), H3K9ac (ab10812, Abcam), H3K4me3 (ab1012, Abcam), H3 (ab1791, Abcam). As controls the following IgGs were used: IgG mouse (sc-2025, Santa Cruz), IgG rabbit (sc-2027, Santa Cruz) and IgG goat (sc-2028, Santa Cruz).

**Primers used for ChIP**

miR144/451 enhancer 5`-GGCTCCTGATAAACAGAAGGCTG-3` and 5`-GGATTGCTGTGAGTAGGGTGTG-3`;

miR144/451 promoter 5`-CCTGGGCTGTGCCTGACCAC-3`and 5`-AGCACTGTGAGGGGCTGGGG-3`or 5`-CCGGTATGATAAAAGGCAAGCAG-3`and 5`-AAGGTTCCCTTTGGTCACACTAA-3`.

**Following primer pair were used for QRT-PCR**

RUNX1

5`- TCGACTCTCAACGGCACCCGA-3`

5`- TGACCGGCGTCGGGGAGTAG-3`

TAL1

5`- TCGGCAGCGGGTTCTTTGGG-3`

5`-CCATCGCTCCCGGCTGTTGG-3`

GATA1

5`- GACACTCCCCAGTCTTTCAGG-3`

5`-CAGTTGAGGCAGGGTAGAGC-3`

Pri-miR144/451

5`-GATGTACTAGTCCGGGCACCCCC-3`

5`-GTGCCCTGGCAGTCAGTAGGTTG-3`

RUNX1/ETO

5`- AAAAGCTTCACTCTGACCAT-3`

5`- TAGTTAACGTTGTCGGTGTA-3`

RUNX1/ETO QRT-PCR Figure 6-7

5`- CACCTACCACAGAGCCATCAAA-3`

5`- ATCCACAGGTGAGTCTGGCATT-3`

GYPA

5`- CCCTCCAGAAGAGGAAACCGGAGA-3`

5`- GGCACGTCTGTGTCAGGTGAGG-3`

GAPDH

5`- TCTTTTGCGTCGCCAGCCGAGC-3`

5`- TGACCAGGCGCCCAATACGACC-3`

TBP

5`- TTCGGAGAGTTCTGGGATTGTA-3`

5`- TGGACTGTTCTTCACTCTTGGC-3`

**Following Taqman probes from Life-Technologies were used:**

hsa-miR-451a (001141), hsa-miR-144-3p (197375_mat), pri-hsa-mir144 (03303762), hsa-RNU6B (001093) and hsa-RNU48 (001006).

**For analyzing mature miRNAs (Figure 1) with the miScript system from Quiagen was used.** Primer assays were as follows:

hsa-RNU6-2 (MS00033740), hsa-SNORD68 (MS00033712), hsa-miR-144-3p (MS00020328), hsa-miR-451a (MS00004242), hsa-miR-126-3p (MS00003430), hsa-miR-27a-3p (MS00003241), hsa-miR-223-3p (MS00003871), hsa-miR-28-3p (MS00009254), hsa-miR-222-3p (MS00007609), hsa-miR-221-3p (MS00003857), hsa-mir-146-5p (MS00003535), hsa-miR-150-5p (MS00003577) and hsa-miR-34a-5p (MS00003318).
